# What is and how do we achieve a resilient digital democracy?

**DOI:** 10.12688/openreseurope.21988.2

**Published:** 2026-02-10

**Authors:** Christian Fuchs

**Affiliations:** 1 Paderborn University Department of Media Studies, Paderborn, North Rhine-Westphalia, 33098, Germany

**Keywords:** resilient digital democracy, resilience, resilience theory, digital democracy, disinformation, fake news, digital authoritarianism, autonomy, digital potestas, digital potentia

## Abstract

This paper develops a new concept and framework for understanding resilient digital democracy in an age defined by polarisation, cascading crises, and the global rise of digital authoritarianism. It begins by tracing the concept of resilience from systems theory to social and political life, showing that resilience in democracy is not a mechanical property of systems but a dynamic, human-driven practice grounded in agency, resistance, and collective self-determination. The paper argues that with emerging challenges in the digital sphere, such as misinformation, surveillance capitalism, platform monopolies, deepfakes, and hybrid warfare, democracy can only endure if its digital dimensions are protected and transformed. Building on this foundation, the paper introduces a holistic approach to resilient digital democracy that spans environmental, technological, economic, political, and cultural domains, and advances strategies such as public-commons digital infrastructures, platform co-operatives, public service Internet platforms, free/libre open source software (FLOSS), participatory innovations, and hybrid offline/online democratic practices. The result is a fresh, interdisciplinary vision of how democracy can be reinvented in the digital age.

## Plain Language Summary

The article asks how democracy can survive in a digital world full of crises, lies, hate, and growing authoritarianism. It says resilience is not a magic system feature, but something people actively build by resisting attacks on democracy and creating fairer rules and institutions.

Digital tools can strengthen democracy – for example through online participation, petitions, citizen assemblies, and protests – but they can also harm it via surveillance, platform monopolies, fake news, deepfakes, and hate speech.

•   The author argues that resilient digital democracy needs:

•   secure, environmentally sustainable digital infrastructures

•   strong laws and protections for rights and privacy

•   more citizen participation and debate, combining online and offline spaces

•   independent, non-profit digital platforms, co-operatives, and open-source software instead of Big Tech control

•   fact-checking and education to spot manipulation.

Overall, a resilient digital democracy uses technology to share power, protect rights, and support active, informed citizens.

## 1. Introduction

This paper poses the following questions: What is a resilient digital democracy? How do we achieve such a type of digital democracy?

It provides an answer by first looking at the origin of the concept of resilience ( Section 2), discussing definitions of resilient democracy ( Section 3), showing that there is not yet a comprehensive understanding of resilient digital democracy ( Section 4), and outlining a novel theoretical concept ( Section 5).

The Global State of Democracy Report 2025 outlines that the year 2025 “underscored the extent to which radical uncertainty remains a defining feature of the global context. The return of Donald Trump to the US presidency reflects this volatility, contributing to the erosion of long-held assumptions across multiple domains – from the economic impacts of tariffs, to the authority of supranational institutions responsible for protecting global peace and stability, to the inviolability of international borders among liberal democracies” ( International Institute for Democracy and Electoral Assistance, 2025, 9). In 2025, press freedom declined in 43 countries, freedom of expression in 39, economic equality in 37, and access to justice in 35 (Ibid., 27: figure 2.6).

Political, economic, and everyday life are not imaginable today without digital technologies. We live in digital societies. The Internet, apps, social media, big data, algorithms, and AI have supported developments that threaten democracy, such as online disinformation, fake news, and post-truth politics, just like they have supported the practicing of relatively novel forms of democracy such as digital participatory democracy, open democratic governance, and democratic cyberprotests. “Half of all autocratizing governments increasingly spread disinformation. Polarization is increasing in a quarter of all countries, often to toxic levels. Mutually reinforcing, they fuel autocratization. Conversely, successful democratizers often reduce disinformation substantially. […] Disinformation, polarization, and autocratization often go hand in hand and mutually reinforce each other – and to some extent the other way around for democratization ” ( V-Dem Institute, 2025, 38).

Today, the world is politically highly polarised. Multiple crises have cascaded into a negative dialectic where global neoliberal capitalism has turned against liberal democracy, which has resulted in the emergence and success of new authoritarian movements. My analysis is here based on the two Frankfurt School philosophers Horkheimer and Adorno (2002), who argue in their book *Dialectic of Enlightenment* that there has been “the self-destruction of enlightenment“, the “enlightenment’s relapse into mythology” (xvi), and that the enlightenment has the tendency of “the reversion of enlightened civilization to barbarism in reality” (xix). They argue that the negative dialectic of enlightenment resulted in Auschwitz, world wars, fascism, and authoritarianism. According to Horkheimer and Adorno, the cause of the negative dialectic of the Enlightenment is that reason was turned into a means of domination and control. Humans are reified and alienated, which means that they become instruments that serve purposes that lie outside of their own interests. Today, we live in a capitalist world system where the economic, environmental, social, political and ideological antagonisms have cascaded into multiple crises that have advanced new forms of authoritarianism, fascism, and nationalism.

In the realm of the public sphere, where citizens inform themselves about and debate politics, a variety of factors have tended to undermine democracy. They include, for example, the digital giants‘big tech monopolies, fake news, deep fakes, misinformation and information manipulation, digital authoritarianism and digital fascism, post-truth culture, online hatred and anti-social social media, digital acceleration that leaves no time for debate, echo chambers and filter bubbles, privacy violations by digital surveillance, AI-generated algorithmic politics that downgrades human action, digital tabloid culture, influencer capitalism, hybrid warfare that includes hacker attacks on digital and media infrastructures, etc. ( Fuchs, 2024).

In the academic literature, one finds a distinction between three processes of autocratisation, namely “democratic regression/backsliding (when the core features of liberal democracy are eroded but no transition to autocratic regime occurs), democratic breakdown (where democracy collapses), or authoritarian deepening (when we observe the deepening of authoritarian characteristics in already autocratic regimes)” ( Tomini, Gibril & Bochev, 2023, 120). Waldner and Lust (2018) review theories that explain the causes of the rise of authoritarianism. They distinguish between “six theory families” explaining autocratisation (109): There are approaches that stress the role of political actors, political culture, political institutions, political economy (income levels, income distribution, macroeconomic development, class relations, etc.), conflicts among groups of citizens, and international actors. Waldner and Lust argue for a model that synthesises such explanations. The six theory families include economic, political, and cultural factors. If a synthesis is feasible, then the implication is that there can be economic, political, and cultural factors that make the rise or strengthening of authoritarianism more likely. In a comparable manner, the political theorist Franz L. Neumann (2017) argues that there are economic, political, and socio-cultural forms of alienation that drive the rise or intensification of authoritarianism if they combine with massive fears among the population (destructive psychological alienation) that turns into persecutory anxiety organised by authoritarian parties, leaders, institutions, or social movements (see also Fuchs, 2017).

Given the rise of new threats to (digital) democracy, such as fake news, Lazer et al. (2018) called for the creation of a “science of fake news”, by which they mean “interdisciplinary research to reduce the spread of fake news and to address the underlying pathologies it has revealed” ( Lazer et al., 2018, 1096). A significant number of such studies has emerged since Lazer et al. made that call. For example, for their book *Network Propaganda. Manipulation, Disinformation, and Radicalization in American Politics,* Benkler, Faris, and Roberts (2018) collected four million online news stories during the 2016 US presidential election. Using computational social science methods, they analyse how these stories were shared on Twitter and Facebook and found that there was “a division between the right and the rest of the media ecosystem. The right wing of the media ecosystem behaves precisely as the echo-chamber models predict – exhibiting high insularity, susceptibility to information cascades, rumor and conspiracy theory, and drift toward more extreme versions of itself” ( Benkler, Faris, and Roberts, 2018, 73). In another study, Vosoughi, Roy, and Aral (2018) collected around 4.5 million tweets from 2006 until 2017 where users tweeted about 126,000 news stories. They found that falsehood “also reached far more people than the truth. Whereas the truth rarely diffused to more than 1000 people, the top 1% of false-news cascades routinely diffused to between 1000 and 100,000 people. Falsehood reached more people at every depth of a cascade than the truth, meaning that many more people retweeted falsehood than they did the truth. […] The greater likelihood of people to re-tweet falsity more than the truth is what drives the spread of false news” ( Vosoughi, Roy, and Aral 2018, 1148, 1150). Such studies of threats to digital democracy have shown that the threats arising from fake news, bots, algorithmic news and politics, and online disinformation campaigngs are serious and real.

Given we are experiencing new forms of authoritarianism, such as digital authoritarianism, the question arises of how we understand authoritarianism. I follow the Frankfurt School-approach for conceptualising authoritarianism (including authors such as Wilhelm Reich, Erich Fromm, Theodor W. Adorno, Max Horkheimer, Herbert Marcuse, and Franz L. Neumann). To understand authoritarianism, Wilhelm Reich argued for an approach that integrates critical psychology, political economy, and ideology critique. In 1936, the Frankfurt School released its study on authority and family. The goal was to understand where and how fascism originated ( Horkheimer, 1936/1987). Fromm defined the authoritarian personality as an individual who “exercises aggression against the defenseless and sympathy with the powerful” ( Fromm, 1936/2020, 24). Fromm characterised the authoritarian personality as necrophilic. He shows that Hitler was a necrophile and that the “necrophile demands annihilation” ( Fromm, 1973/1997, 406), that Stalin was a mental and physical sadist, and that both stand for human destructiveness that is characteristic for authoritarianism that is opposed to humanism.

Authoritarianism includes top-down-leadership structures, nationalism, the friend/enemy-scheme, and repression/violence ( Fuchs, 2022a). Authoritarians define outsiders and scapegoats who are blamed for the ills of society and social systems and against whom they propagate the use of repressive or even violent means. The advantage of the notion of authoritarianism, as utilised by the Frankfurt School, is that it is neither micro nor macro, but micro, meso, and macro: Authoritarianism and fascism are not organised at single levels, but take place at encapsulated levels where each level presupposes and goes beyond the underlying level: the individual, the group, social movements/parties, social systems/Institutions, and society ( Fuchs, 2022a, 2024).

Fascism is a particular, radical form of authoritarianism. Its central feature is that it favours, propagates, uses, and institutionalises violence as the main political means and organisational principle of society. Violence is the fascists’ ultima ratio. The fascist “cannot help feeling surrounded by traitors, and so continuously threatens to exterminate them” ( Adorno, 1975, 78). In fascism, politics “grows out of the barrel of a gun” ( Arendt, 1970, 37), while in a democracy, politics grows out of the “generative force of communicative freedom” ( Habermas, 1996, 151). “Terror becomes total when it becomes independent of all opposition; it rules supreme when nobody any longer stands in ist way. If lawfulness is the essence of non-tyrannical government and lawlessness is the essence of tyranny, then terror is the essence of totalitarian domination” ( Arendt, 1951/1973, 464).

Arendt (1951/1973) draws a distinction between authoritarianism and totalitarianism: “authority, no matter in what form, always is meant to restrict or limit freedom, but never to abolish it. Totalitarian domination, however, aims at abolishing freedom, even at eliminating human spontaneity in general, and by no means at a restriction of freedom no matter how tyrannical” (405). While for Arendt the problem is totalitarianism, for the Frankfurt School it is authoritarianism. Arendt sees totalitarianism as rooted in political breakdown (state, law, parties, citizenship). She gives a political explanation of it. In contrast, for the Frankfurt School authoritarianism arises from the administered world, a world that is ruled by instrumental reason.

Max Horkheimer (1939/1989, 78) noted that “whoever is not willing to talk about capitalism should also keep quiet about fascism”. Given that today Silicon Valley as capital’s hegemonic faction supports authoritarianism, we, just like at the time of Horkheimer, also today need a focus on the analysis of the interconnection of (digital) capitalism and (digital) fascism. Discussions about resilient (digital) democracy stand in the context of the rise of new forms of authoritarianism and fascism.

Digital technologies have become an important means of digital authoritarianism that tries to undermine and abolish democracy ( Maerz, 2024). In this context, digital democracy includes practices that, supported by digital technologies, foster a variety of democratic models that challenge and work against digital authoritarianism. Digital democracy includes a variety of practices, including, but not limited to, digital participatory budgeting, democratic cyber-protest and cyber-activism, online petitions, political online debates, e-voting, online public consultations, digital town halls, online citizen assemblies and mini-publics, etc. Resilient digital democracy has to do with the question of how digital democracy can be sustained and defended against authoritarian and fascist developments.

In the light of such developments, the European Union has introduced the development of the European Democracy Shield in order to defend democracy. In this context, the notions of resilient democracy and democratic resilience have gained prominence. The EU sets up a European Centre for Democratic Resilience in order to “withstand evolving common threats, in particular foreign information manipulation (FIMI) and disinformation” ( European Commission, 2025, 2–3). The challenges democracy faces include “rising extremism and polarisation, declining trust and engagement, threats to the integrity of elections and the plurality of public debate and free speech, and a deterioration of the environment in which journalists and civil society operate. These challenges come amidst a deep digital transformation of our societies, which has reshaped the way in which public debate takes place, how information flows and how citizens engage in the public sphere. This has brought significant new opportunities for people to freely access information, express their opinion and participate in public life and democratic debate” ( European Commission, 2025, 1).

The EU’s focus makes clear that making democracy resilient has a great deal to do with (dis) information that is circulated and reaches the public sphere via digital media. In digital societies, we cannot separate democracy from digital spaces; democracy is not only but also always digital democracy. Wanting to make democracy resilient against authoritarianism, therefore, implies that we must make digital democracy resilient against digital authoritarianism. To do so, we need to start with a couple of very basic questions. We need to ask: What is a resilient digital democracy? What is resilience? What are the specific features of resilience in the context of digital democracy? This paper addresses these questions.

## 2. Resilience

Resilience is a concept that emerged in systems theory. C. S. Holling is the most influential theorist of resilience. He defines resilience as “the persistence of relationships within a system and is a measure of the ability of these systems to absorb changes of state variables, driving variables, and parameters, and still persist. In this definition resilience is the property of the system and persistence or probability of extinction is the result” ( Holling, 1973, 17). For Holling, resilience means the capacity of a system to survive crises, disruptions, shocks, and attacks. Consequently, democratic resilience in the systems theoretical categories established by Holling and others means that democracies are capable of surviving societal crises, disruptions, shocks, and attacks. The rise of authoritarianism and a fascist coup d’état are examples of democratic crises that pose the threat of the end of democracy. In the light of such crises, the question arises of how resilient democracies are or how they can be made more resilient. For the philosopher of information Wolfgang Hofkirchner, resilience means “stability in pure living systems that are able to react more flexibly than pure material systems, to bounce back after disturbances, to recover, to repair themselves” ( Hofkirchner, 2023, 142).

For Holling (1973), resilience is a feature of ecosystems, for Hofkirchner (2023), one of living and social systems. However, it is not clear why resilience should not be a capacity of physical systems, too. For example, natural rivers are often able to show resilience against heavy rainfall and storms that, as disturbances, threaten to result in overflowing and breaking down the river’s flow pattern. Heavy rainfall increases the volume of water in the river as well as the speed of the water flow. The river’s meanders, its curves and bends, slow down the speed, so it has a certain resilient capacity. In addition, a river has the capacity to move sediment such as sand and gravel downstream and deposit it on land, which builds new land that acts as a barrier against flooding. Meanders and sediment are the river’s two mechanisms of resilience. The straightening of rivers, for its industrial use as a transport system so that ships transport goods, commodities, and people in an easier and faster way, decreases its capacity for resilience. Straightened rivers are much more prone to flooding. In addition, residential areas have often been built close to rivers as part of urban expansion, access to water, and the organisation of transportation and trade. As a consequence, the decreased resilience of rivers to flooding has, in the context of urbanisation and industrialisation, increased the risk that humans are negatively affected.

Resilience is not just a capacity of living and social systems but a general capacity of systems. That a system shows resilience means in the language of systems theory, that in a situation of heavy disturbance and chaos, the system does not break down but continues to maintain itself, which means that its elements, the relations between the elements (the system’s structure), the system boundary the delimits the system from its environment, and the system’s dynamic (the behaviour that the elements in relation to each other show as reaction to inputs) continue to exist.

Having established what resilience is all about, we need to next ask what a resilient democracy is.

## 3. Resilient democracy

Society differs from physical and living systems: Humans are societal, self-conscious and social beings. They can anticipate the potential effects of their actions and potential future states of society, which enables them to choose between different actions that they take or do not take. Humans are consciously and socially producing beings ( Fuchs, 2020, chapters 3 & 4). They form moral values, norms, judgments. Humans in social relations produce and reproduce social structures (resources and rules) that enable and constrain their actions (see Giddens, 1984, 25). In society, there is a dialectic of social production and communication: Humans communicate in order to organise production, and they coordinate production through communication. Communication is humans’ production of social relations through symbolic interaction. It is a special (symbolic) type of production. Social production is based on and requires communication as one of its necessary but not sufficient conditions.

Resilience in society differs from resilience in other systems in that humans are moral beings who make judgments about what they see as desirable and undesirable and have the capacity to plan and take actions in order to try to avert undesirable developments of society and the social systems they live in. In society, resilience is not simply a feature of the social system because it is humans and not society that act consciously, actively, socially, and morally. Resilience in society means that humans act in ways that resist or build structures that support them in resisting developments of social systems or society in directions that they consider undesirable. As an act of resistance, resilient action is always a political and normative form of action. But neither resistance nor resilience are automatically democratic. For example, a fascist system can use militarism as a form of resilience that allows the maintenance of dictatorship by violently repressing any signs of political opposition. It is therefore important to talk about resilient democracy as a particular type of social resilience.

Let us have a look at some definitions of democratic resilience and resilient democracy:

(a) Burnell and Calvert (1999, 4) understand the resilience of democracy as an “attachment to democratic ideals […], in spite of hostility from the officially prescribed values and norms and apparent indifference from many elements in society. The persistence of the faith can be evident even where the institutional forms have been lost. […] Persistence, durability and resilience are not one but several conditions, and taken together they make up what can be called ‘perseverance despite‘”.
(b) Democratic resilience means “the ability to prevent substantial regression in the quality of democratic institutions and practices” ( Boese *et al.*, 2021, 886).
(c) “we define democratic resilience as the persistence of democratic institutions and practices, despite the existence of political forces that implicitly or explicitly attack the liberal democratic regime. In other words, democratic resilience means the ability to avoid the onset of autocratization as well as attempts of regime breakdown” ( Meléndez & Kaltwasser, 2021, 955).
(d) “ *Democratic resilience is the ability of a political regime to prevent or react to challenges without losing its democratic character.* […] Democratic resilience is the ability of a democratic system, its institutions, political actors, and citizens to prevent or react to external and internal challenges, stresses, and assaults through one or more of the three potential reactions: to withstand without changes, to adapt through internal changes, and to recover without losing the democratic character of its regime and its constitutive core institutions, organizations, and processes. The more resilient democracies are on all four levels of the political system (political community, institutions, actors, citizens) the less vulnerable they turn out to be in the present and future” ( Merkel & Lührmann, 2021, 872, 874).
(e) “We thus define ‘democratic resilience‘broadly as a system’s capacity to withstand a major shock such as the onset of extreme polarization and to continue to perform the basic functions of democratic governance – electoral accountability, representation, effective restraints on excessive or concentrated power, and collective decision-making” ( Lieberman *et al.*, 2022, 7).
(f ) “Democratic resilience is the capacity of a democratic regime to absorb external challenges and internal stressors and to dynamically adapt to the changing functional conditions of democratic governance without falling into regime change and abandoning or damaging democracy`s defining principles, functions and norms” ( Merkel, 2023, 3).
(g) Democratic resilience is “the *ability to maintain democratic governance functions and principles, despite attempts by illiberal actors to damage or diminish vertical, horizontal, or diagonal accountability mechanisms that are core to democracy*” ( Shein *et al.*, 2023, 11).
(h) The International Institute for Democracy and Electoral Assistance publishes reports on the global state of democracy, where democracy’s resilience plays an important role. It understands democracy’s resilience as democracy’s “ability as a political system to recover, adapt and/or flexibly address such complex challenges and crises” ( International Institute for Democracy and Electoral Assistance, 2017, xiv), “to cope with, innovate, survive and recover from complex challenges and crises that present stress or pressure that can lead to systemic failure” (xiv). “Resilient social systems are flexible (able to absorb stress or pressure), can recover from challenges or crises, adaptable (can change in response to a stress to the system), and innovative (able to change in order to more efficiently or effectively addresses the challenge or crisis)” ( International Institute for Democracy and Electoral Assistance, 2017, 38; see also Sisk, 2017).
(i) Walz *et al.* (2025, 100) understand democratic resilience as “a democratic system’s capacity to prevent, cope with (either by withstanding or adapting) or recover from stressors challenging democratic quality and persistence without losing its democratic character“.
(j) Croissant and Lott (2025, 3–4) define democratic resilience as the “abilities of a political system that help democratic procedures, principles and practices to maintain their given achievement levels, to resist erosion or breakdown and to develop sufficient elasticity to bounce back from regress. […] If democracies are resilient, they are not only able to fend off stressors and crises, but also to develop coping mechanisms allowing them to deal with risks in the long run, preserving, reviving or enhancing their level of ‘democraticness‘“. The authors argue that political resilience includes both state and non-state actors, namely political institutions, political parties, civil society, and the political community (5).
(k) “Democratic resilience is the capacity to withstand, adapt to, or recover from shocks and stressors without losing democratic quality” ( Riedl *et al.*, 2025, 167).

In definition (a), resilient democracy is purely conceived in subjective terms, a form of consciousness, moral value, and faith to which some humans remain dedicated even when democratic institutions are threatened or have been abolished. The definitions (b-k) conceive of resilient democracy in systemic terms. This becomes evident by sentences that define resilience as the “ability of a political regime”, the “ability of a democratic system”, “a system’s capacity”, or the “capacity of a democratic regime”. The authors of such definitions conceive of resilience as systemic, that is, as a property of social systems. They see it as a feature, ability, and capacity of the democratic system, namely as the ability of the democratic system to prevent its own breakdown and transition into authoritarianism. In such definitions, political and democratic systems are the actors. These systems, not humans, are presented as doing something in order to strengthen resilience as the system’s capacity.

Some works argue that resilience “legitimates a neoliberal model of development based upon the constitution of markets and the interpellation of subjects within markets” ( Reid, 2012, 74). Banaji (2024) argues that resilience is a neoliberal ideology that has a focus on “internal resistance” (144) of communities against violence that lacks a focus on solidarity. The concept would only look at those “who appear to survive and thrive despite the pressure of social intolerance and hate” (152) while forgetting those “who fought and died, or fought and were imprisoned or fought and lost their families or spoke out and lost their jobs and fell into depression are simply relegated and forgotten” (147).

Conceiving of resilience as a systemic feature that is autopoietic and independent from the system’s environment certainly poses the dangers that critics outline. In reality, systems are, however, never closed but always interact with their environments. For example, that a certain local community is resilient against fascism does not tell us how that condition has been achieved. There might have been national legislation that outlaws certain fascist groups and socio-economic policies that improved the living conditions throughout the country, so that fascism lost its material ground, etc. These are measures that are external to the local community. At the same time, a citizen movement in the local community may have organised itself in order to campaign against fascist movements. What I want to say is that resilience has many causes; it is a complex phenomenon. Reducing it to internal systemic dynamics can indeed turn it into an ideology. But resilience can also be conceived of as practices of resistance that create structures and have both internal and external drivers.

Along these lines, after reviewing 58 definitions of resilient democracy, Holloway and Manwaring (2023, 73) argue that “a resilient democratic system likely results from both citizens and the state. There is a role for the state in, at least, ensuring effective institutions and readiness for change, promoting equity and diversity, creating opportunities for inclusive participation, and facilitating learning from system shocks“. David Chandler adds that resilience thinking “provides a new role for government and justifies new levels of societal intervention and the development of the professional and ethical expertise necessary for this task” ( Chandler, 2014, 226).

The authors of the definitions (b-k) take a structuralist-functionalist position in defining resilience. They understand resilience as the ability of the political system to preserve democracy and to prevent autocracy. In contrast to such definitions, the present author takes an actor- and action-based human-oriented approach that stresses that in society, only humans and not social systems are actors. Taking such a focus means that resilient democracy is not a capacity of the political system but a social action of humans who resist authoritarianism or create or reproduce structures that support resistance against authoritarianism and the emergence of dictatorship. Resilient democracy is a process where democrats produce and reproduce structures of democratic resilience that condition democratic action that again reproduce and produce structures of democratic resilience.

An action-based humanist approach faces the question if autonomous AI-based system such as self-driving cars, autonomous weapon systems, cleaning robots, generative AI systems such as ChatGPT, or Internet bots are actors or not. From a humanist perspective, the answer is that so-called autonomous technologies do not operate independently from humans but depend on them. They depend on human engineering, programming, repair, energy produced by humans, etc. In addition, they do not have the same features as humans have, which is why robots and AI cannot replace humans. “Robots cannot truly replace humans because they lack praxis and poiesis, which means they do not understand what it means to be social, what it means to love, what it means to be sad, what morality is, what desires and creativity are etc. They lack fundamental human capacities. Robots do not have feelings. They do not have intentions. They do not have morals. They do not have desires. They do not have a will. They do not understand and experience love, sadness and happiness. Computers cannot deal with ambiguity and dialectics. They operate based on algorithms, while humans are non-algorithmic” ( Fuchs, 2022b, 146-147). The philosopher Hubert Dreyfus (1986, xiv) argues in this context: “Human emotional life remains unique, to be sure, but what is more important is our ability to recognize, to synthesize, to intuit. There are good reasons to believe that those abilities as well are rooted in processes altogether different from the calculative reason of computer programs”.

Rejecting functionalism and structuralism, the socialist theorist Anthony Giddens speaks of the duality of structure: “The social systems in which structure is recursively implicated, on the contrary, comprise the situated activities of human agents, reproduced across time and space. […] According to the notion of the duality of structure, the structural properties of social systems are both medium and outcome of the practices they recursively organize” ( Giddens, 1984, 25). In the context of resilient democracy, duality of structure means that there are capacities, actions, and structures that allow democracy to be reproduced across time and space, withstand crises, and resist authoritarianism. Democrats produce structures of resilient democracy that are both medium and outcome of democratic practices. Merkel (2023) argues that resilient democracy refers to the resilience of democracy’s core institutions (constitution, party system, political culture), political parties, civil society, and political community. He includes both structural features, namely democratic institutions, as well as actors such as parties, NGOs, and citizens. Merkel’s approach indicates that one needs to think about both structures and actors when talking about resilience.

In the academic literature, one can find the expressions “democratic resilience”, “resilient democracy”, and “resilience of democracy”. They tend to be used interchangeably. However, strictly speaking, democratic resilience is different from resilient democracy and the resilience of democracy. The latter two terms imply that resilience is a particular potential feature of democracy. In contrast, the first indicates that there are democratic and undemocratic forms of resilience, so democratic resilience refers to the organisational mode of resilience. What most approaches actually want to describe is not a feature of resilience (democracy as a feature and property of resilience) but a feature of democracy (resilience as a feature of democracy). Democratic resilience is an organisational mode of resilience. There can be potentially other non-democratic forms of organising resilience. In this paper, we are interested in how digital democracy can be made resilient and not so much in resilience’s organizational mode, which is why therefore we avoid using the term “democratic resilience” and prefer to speak of “resilient democracy” and “the resilience of democracy”.

Democracy is many things at the same time. Democracy is an idea. Democracy is a set of principles (“democratic”). Democracy is a group of people (“citzens”, “democrats”). Democracy is a type of action (“democratic practices”, “struggles for democracy”, “democratising”, etc.). Democracy is an institution and system (“democracy”). Democracy is dynamic, not static. It is a processes, in which humans, based on constitutional principles, engage in political actions and participate in collective decision-making so that collective decisions (rules, policies, laws) emerge that represent the collective will of those participating. Democracy means that those who live together in a society or social system share the collective right to participate in collective decision making, that is, in making decisions that affect their lives. They do so through mechanisms such as public debate, voting. As a consequence, democracy is a political model of distributed power opposed to models where power is controlled by a few. There is not one understanding and model of democracy. Rather, there is a variety of models and dimensions of democracy (see Fuchs, 2025b). They include: participatory democracy, deliberative democracy, constitutional democracy, representative democracy, direct democracy, and pluralist democracy. An actual democratic system often combines various models of democracy. Democracy is opposed to other political systems, especially dictatorship, which is a system where decision-making is the right and domain of a single person or group. Some typologies include oligarchy as a third type of governance and conceive of oligarchy as a system where we find the rule of the few. Oligarchy is in such classifications opposed to the rule of the one (dictatorship) and the rule of the many (democracy) (for example, Aristotle & Lord, 2013, 73).

The continuum of politics between democracy and dictatorship plays an important role in resilient democracy. Building on the definitions outlined above, we can understand and define democracy’s resilience as actors in society, such as citizens, governments, political parties, social movements, or non-governmental organisations, creating mechanisms that resist the transformation of society from a democracy into a dictatorship or actively and successfully resisting such a transformation. Resilient democracy has a subjective and an objective dimension; it is both actor-and systems-based and involves both the subjective features of practices and ideas as well as objective structures. Resilient democracy requires humans committed to democracy who, as democratic actors, create democratic structures and mechanisms that resist dictatorships and dictatorial tendencies and try to institutionally shield democracy against its breakdown and transition into dictatorship. Actors in resilient democracy involve institutions, parties, citizens, and civil society/social movements. That a democracy is resilient does not simply mean that it remains stable. Holloway and Manwaring (2023, 73) point out the dynamic character of resilient democracies, namely that such resilience can involve the “persistence, adaptation, and/or transformation” of democracy.

In the approach of Boese *et al.* (2021), there are two stages of resilient democracy: onset resilience (the prevention of autocratisation before its onset) and breakdown resilience (the avoidance of the breakdown of democracy after the start of autocratisation) ( Boese *et al.*, 2021, 886). For Boese *et al*., democratic resilience is a two-stage process: “In the first stage, democracies exhibit resilience by maintaining or improving their level of democracy. Put differently, first-stage resilient democracies avoid the onset of autocratization. For this reason, we refer to the first stage as onset resilience. In the second stage, democracies that are experiencing autocratization can demonstrate resilience by averting democratic breakdown. This second stage of democratic resilience thus involves avoiding a regime change. We refer to this second stage as breakdown resilience” ( Boese *et al.*, 2021, 887). Onset resilience means that the transition from democracy to authoritarianism is halted in an early stage of the latter’s emergence. Breakdown resilience means that authoritarian movements and tendencies have already developed well, the transition from democracy to dictatorship has become likely, and such a transition can be averted. Boese *et al.*’s (2021) two stages have to do with the temporal dimension of democratic resilience.

The rise and strengthening of authoritarian movements, which pose the danger of the emergence of a dictatorial society, is often the consequence of a large-scale crisis or an interaction and reinforcement of various crises. Crises have a variety of causes. Based on a typology of society’s subsystems ( Fuchs, 2020; Hofkirchner, 2002), we can distinguish between environmental, technological, economic, political, and cultural crises (see Table 1). Hofkirchner (2002, 2006) adds that society consists of the sociosphere, the technosphere, and the ecosphere. He divides the sociosphere into the economy, the political system, and culture. Crisis and multiple crises are situations where society enters a state of high uncertainty, so that the future is put into question. The outcome is always open and depends on collective human action. If authoritarian movements are strengthened in crises, then the breakdown of democracy becomes more likely. Therefore, resilient democracy especially matters in situations of multiple crises. Crises turn into a crisis of society when there is a large-scale disruption of life as a consequence of crises, so that society as we know it is threatened with complete breakdown. A societal crisis can be the consequence of a) global problems such as the climate crisis, large-scale environmental pollution, natural disasters, wars, etc., that destroy society’s infrastructures that humans require to survive or b) uprising, protests, or protest behaviour (including “protest votes”) that are due to the large-scale dissatisfaction of citizens. When authoritarian movements become strong, a crisis of society can turn into a “fascism-producing crisis” ( Eley, 2015, 112).

**Table 1.** A typology of crises.

Subsystem of society	Type of crisis	Definition
environment	environmental crisis	the breakdown of the natural systems and processes that sustain life
technology	technological crisis	The breakdown of technological infrastructures that humans depend on to carry out essential activities, such as communication, transportation, energy production, data storage, healthcare operations, or financial transactions.
economy	economic crisis	the breakdown of economic organisations, which causes unemployment and an increase in poverty, precarity, socio-economic inequality, and despair
political system	political crisis	the breakdown of government or the trust of citizens in government or the political system due to political developments such as corruption, misgovernment, or policies that cause large-scale dissatisfaction, warfare, terrorism, organised crime
culture	cultural crisis	the breakdown of dominant norms, ideas, lifestyles, customs, and forms of meaning-making, health risks such as pandemics
society	societal crisis	the breakdown of society as such or society as one knows it

**Table 2.** Processes that disrupt digital democracy.

Subsystem of society	Type of disruption	Definition
environment	environmental disruption	environmental crises result in natural disasters that destroy technological and social infrastructures required to run digital platforms, including digital democracy platforms
technology	technological disruption	hacking that aims at destroying the digital hardware, software and content that digital democracy requires
economy	economic disruption	economic monopoly of anti-democratic platforms, lack of support and funding for digital democracy platforms and projects
political system	political disruption	censorship and outlawing of digital democracy, surveillance of digital democracy platforms, use of violence against the operators of digital democracy projects or the users of digital democracy platforms, hybrid warfare and terror that attacks digital infrastructures, cyber-crime
culture	cultural-ideological disruption	fake news and deep fake campaigns, hate speech, disinformation, post-truth culture, online echo chambers, digital tabloidisation
society	societal disruption	One or several disrupting factors cause the breakdown of not just single platforms, but (digital) democracy as a whole, so that (digital) authoritarianism dominates

What is a crisis? “A crisis is a moment when there is the possibility of large-scale change consequent upon a small event in a narrow window of time. The lack of proportionality between cause and consequence is inherent to the definition of crisis. Crises differ in their consequences. There are four main types: the crisis leads to system breakdown; after the crisis there is a return to pre-crisis conditions; the crisis drives the renewal of the system along its existing path of development; or the crisis leads to a new type of system. System breakdown, meaning catastrophe or disaster, is the threat that lurks behind the concept of crisis” ( Walby, 2015, 34).

Having discussed the relationship of resilience and democracy, we will next shift the analysis to the realm of digital media and communication. For doing so, we will in the next section have a look at how in academic works, resilience has thus far been discussed in the context of digital media.

## 4. Resilience and digital media

This section briefly discusses existing academic literature on digital democracy’s resilience. Thus far, this topic has hardly been addressed. In November 2025, a title search using the keywords resilien* AND digital AND democra* resulted in one publication indexed in Web of Science and two indexed in Scopus ^1^. Thus far, the number of academic publications on resilient digital democracy is very small.

Some work has been done on the more general topic of digital resilience. The authors of the book *Digital Resilience: Navigating Disruption and Safeguarding Data Privacy* provide the following definition: Digital resilience is the “organization’s or individual’s ability to maintain the confidentiality, integrity, and availability of crucial information and systems, achieved through a cohesive integration of technical, operational, and organizational actions while withstanding persistent threats, by implementing robust cybersecurity controls, effective incident response strategies, and a culture promoting cybersecurity awareness and education, all while recognizing the inevitability of security incidents and necessitating rapid and efficient counteractions, is defined as ‘digital resilience’” ( Shandilya *et al.*, 2024, 10). Barojan (2021, 69) provides a comparable definition: “Digital resilience refers to boosting digital communities’ ability to cope with internal and external challenges in the information domain“. Another work defines digital resilience in the following manner: “ *Digital resilience refers to the capabilities developed through the use of digital technologies to absorb major shocks, adapt to disruptions, and transform to a new stable state*” ( Boh *et al.*, 2023, 344). Such works on digital resilience often do not have a particular focus or interest in digital democracy’s resilience.

These three definitions of the same term differ. The third one refers to crisis management with the help of digital technologies. An example application is the use of digital media as a means of communication in the COVID-19 pandemic for the continuation of work, friendships, the public sphere, etc. Kaufmann (2017, 115) speaks in this context of the digitisation of resilience, by which she means “the increased circulation of digital information during emergencies” and that “big data analysis has become an important aspect of resilience management”. In contrast, the first and second definitions understand digital resilience as the resilience of digital infrastructures. Measures that are stressed for advancing digital resilience are technological (cybersecurity) and cultural (education) in character. What is missing are political economy measures having to do with economic resources, ownership, labour organisation, policies, laws, etc. The discussion shows that in the academic literature, a distinction has been drawn between the resilience of the digital and the digitalisation of resilience. Both processes have been subsumed under the concept of “digital resilience”.

Some other works have more directly focused on the relationship between resilience and digital democracy. Frischlich and Humprecht (2021) discuss measures that are required to make democracy resilient against online misinformation, including trustworthy behaviour of politicians, legacy media, the creation of a shared identity among different social groups, and individual skills to “identify trustworthy information“ and “check information encountered online” (5). Although the report holds the title “Trust, Democratic Resilience, and the Infodemic“and deals with misinformation spread online and on social media, no definition of digital resilience or digital democracy’s resilience is provided. The question of digital democracy’s resilience is not posed.

Analysing the use of digital democracy tools during the COVID-19 pandemic in Latin America, Lima says that digital democratic innovations are a way of making democracy more resilient. The author argues that “the resilience of democratic systems is significantly reinforced by digital platforms, which facilitate sustained citizen participation and the continual adaptation of democratic practices. E-participation contributes to democratic resilience by enabling continuous citizen engagement, particularly during crises that risk hindering traditional participation methods” ( Lima, 2025, 4). Digital platforms do not just foster democracy but can also, in the form of online fake news, online hate speech, and online echo chambers, support the undermining of democracy. Lima’s paper focuses on digital technologies as a tool for advancing democracy’s resilience, which subsumes the digital under the resilience of democracy. Foregrounding the digital in contrast means to ask how digital democracy can best be made resilient, which is different from asking how digital tools can make democracy resilient.

Studies such as the ones by Frischlich/Humprecht and Lima pose interesting questions about what roles digital technologies can play in making democracy resilient. They subsume the digital under resilient democracy. Weinhardt *et al.* (2024, 131–132) summarise the definition of the use of digital technologies for advancing democracy in the following manner (they speak of “resilient digital democracies”): “By promoting principles such as transparency, accountability, and inclusiveness, the objective is to ensure that digital platforms not only serve economic and functional purposes but also contribute positively to social and societal well-being and the democratic experience”.

Another important question is what resilient digital democracies are, that is, what needs to be done to protect digital democracy against turning into digital authoritarianism or digital fascism. This is the question that this paper addresses. The next section deepens this analysis.

## 5. Resilient digital democracy

Digital democracy means the mediation of democracy by digital information and communication technologies. “Digital democracy is the use of digital media in the practice of democracy. It refers to the digital mediation of democracy. Digital democracy is the mediation of democracy by a variety of digital media, including, but not limited to, the Internet, the WWW, smartphones, apps, tablets, social media, online discussion forums, electronic voting systems, computer-supported co-operative work technologies, artificial intelligence technologies, audio and video streaming platforms, podcasts, etc. […] The features and affordances of digital technology use, such as convergence and presumption (productive consumption), shape the practices and models of digital democracy. Digital democracy has three encapsulated symbolic dimensions: the production and sharing of democratic information (digital democratic information), democratic communication (digital democratic communication), and the co-creation of democracy (digital democratic co-production)” ( Fuchs 2025b, 88-89). Reviewing the state of the art of digital democracy theory and research, Fuchs (2025b) identifies six models of digital democracy: constitutional digital democracy, deliberative digital democracy, participatory digital democracy, representative digital democracy, direct digital democracy, and pluralist digital democracy. Digital democracy does not imply the pure practicing of politics online. Rather, in many instances, digital democracy will work better if it is organised in hybrid offline/online forms, where humans encounter each other personally and face-to-face offline, which is a better-suited way of building trust and starting political debates than online spaces, and digital media are used as supportive but not exclusive means of communication.

Digital democracy can be disrupted by environmental, technological, economic, political, and cultural-ideological processes, or a combination of such factors. The next table gives an overview of processes that disrupt digital democracy.

The question now arises: what actions can be taken to make digital democracy resilient against disruption? Table 3 provides an overview of strategies for making digital democracy resilient.

**Table 3.** Strategies for resilient digital democracy.

Subsystem of society	Type of disruption	Definition
environment	environmental disruption	decentralised data storage, backups, data minimisation, green computing
technology	technological disruption	investment into the development of advanced digital security technologies, employment of well-paid and highly skilled computer scientists, as well as data protection and security professionals
economy	economic disruption	democratic regulation of the digital giants (big tech); legal, institutional, and financial support of fact-checking; highly-skilled and well-paid fact-checkers; using, developing, and supporting platform co-operatives, public service Internet platforms, as well as free, libre and open source (FLOSS) software in the context of digital democracy
political system	political disruption	constitutional safeguards of (digital) democracy, support of democratic civil society organisations, (digital) democratic activism and resistance against authoritarianism; supplementing representative democracy by participatory and deliberative (digital) democracy
culture	cultural-ideological disruption	democratic regulation of the digital giants (big tech); legal, institutional, and financial support of fact-checking; highly-skilled and well-paid fact-checkers; using, developing, and supporting platform co-operatives, public service Internet platforms, as well as free, libre and open source (FLOSS) software in the context of digital democracy
society	societal disruption	a combination of measures from the other dimensions of resilient digital democracy

*First*, there are *environmental* aspects of *resilient digital democracy.* Natural disasters can physically destroy digital infrastructures. One strategy against this threat is decentralised data storage with a significant number of backups. Digital platforms require energy as a power source. They consume significant volumes of energy. Given that the majority of the world's energy production is based on fossil fuels and not on green energies, energy-intensive data centres contribute to the deepening of the climate crisis. The world’s dominant Internet platforms store big data about users in order to sell personalised ads. Digital democracy apps and platforms can make a difference and become more environmentally resilient and sustainable by minimising the storage of data so that only data that is necessary for guaranteeing the technologies’ functioning is stored. A prerequisite for doing so is that digital democracy platforms take on a non-commercial character so that they do not have an incentive to gather and process vast amounts of data and meta-data. In addition, building digital democracy platforms as far as possible on renewable, green computing hardware is a feasible measure. Data minimisation and green computing are strategies for reducing the contribution of digital democracy platforms to environmental crises that, in turn, can disrupt digital platforms, including digital democracy platforms.

*Second*, there *are technological* aspects of *resilient digital democracy.* Hacker attacks on servers, platforms, content, data, and meta-data are attempts to technologically disrupt digital democracy. The best measure that can be taken is that digital democracy projects, public administration, and government continuously invest in the development of advanced digital security technologies and employ well-paid and highly skilled computer scientists and data protection and security professionals.

*Third*, there are *political* aspects of *resilient digital democracy.* Political disruption means that digital democracy platforms are censored, monitored, outlawed, or that violence is used against digital democracy operators or users. In a democratic society, strong constitutional guarantees against the censorship, monitoring, outlawing of (digital) democracy and any other forms of repression against (digital) democrats can support (digital) democracy’s political resilience. Citizens’ financial and personal support of human rights organisations, consumer protection organisations, as well as privacy and digital rights proponents, is another measure for advancing (digital) democracy’s political resilience. In authoritarian societies and societies where democracy is at risk of turning into authoritarianism or where democracy is step-by-step undermined so that there is a creeping form of authoritarianism, (digital) democratic activism and resistance are the best measures for struggling against authoritarianism. Such activities include, for example, protest movements, street protests, political campaigns, civic activism, civil disobedience, digital activism, and the development and use of liberation technologies.

Citizens support authoritarianism because they feel alienated in multiple ways, economically, politically, culturally, and socially ( Neumann, 1957/2017). Political alienation means that citizens feel politics is too distant from them and that political representatives often do not realise what they promise to do, so that there is a gap between promises and reality. Political alienation is one of the factors that contribute to the crisis of democracy. Democracy today is predominantly a representative democracy, so the crisis of democracy is a crisis of representative democracy. Renewing democracy and making it resilient requires institutional reforms. One possibility is to open up representative democracy and combine it with elements of participatory and deliberative democracy under the premise of constitutional democracy. Digital democracy is particularly suited for practising participatory and deliberative democracy ( Elstub & Escobar, 2019, 16; Hennen *et al.*, 2020; Lindner & Aichholzer, 2020, 20). Digital democracy projects can therefore help to renew democracy and supplement representation by participation and deliberation. A good starting point is communal and local democracy, where citizen participation can take place in the form of (digital) participatory budgeting, (digital) citizen juries, and (digital) citizen assemblies/forums. Digital democracy does not mean the practice of democracy purely online. Rather, it requires hybrid online/offline formats where citizens build trust offline and continue the conversations online. Starting at the local level, digital technologies that engage citizens in conversations, policy consultations, policy recommendations, and decision-making can help make representative democracy more communicative and participatory.

*Fourth*, there are *economic* aspects of *resilient digital democracy.* They are closely related to the *cultural aspects of resilient digital democracy* that form the *fifth dimension.* Digital democracy is economically disrupted when platforms that advance and support authoritarianism dominate the public sphere, or there is a lack of funding. The online spreading of fake news, deep fakes, hate speech, disinformation and post-truth culture, the formation of online echo chambers, and digital tabloidisation (a strong entertainment focus that displaces political information and communication, sensationalism, scandalisation, personalisation, simplification, emotionalisation, superficial and brief news disseminated news, digital acceleration and high speed online culture, etc.) are means of disseminating anti-democratic ideology online that disrupts digital democracy at the cultural level.

An example of the intersection of economic, political and ideological power in global digital capitalism is that Silicon Valley big tech gurus such as Elon Musk, Peter Thiel, Marc Andreessen, Mark Zuckerberg, etc. have supported Donald Trump. After buying Twitter and turning it into X, Elon Musk got rid of fact-checking and the ban of extremist accounts. In January 2025 when Trump started his second presidential term, Zuckerberg ended Meta’s fact-checking programme. He characterised fact-checking as “too politically biased” ( Zuckerberg, 2025). Fact-checkers are professional journalists who know how to check if certain claims are true or false. They are part of a news culture that works for free expression and against political bias. Critics argued that the decision effectively benefits far-right disinformation ecosystems by reducing moderation capacity. On 4 September 2025, Donald Trump hosted a dinner with top Silicon Valley big tech CEOs at the White House. They included Sam Altman (OpenAI: ChatGPT), Tim Cook (Apple), Bill Gates (Microsoft), Safra Katz (Oracle), Sundar Pichai (Alphabet: Google, YouTube), and Mark Zuckerberg (Meta: Facebook, Instagram, WhatsApp). An iconic video ( https://www.youtube.com/watch?v=dsl_sKYywEI, accessed on 30 January 2025) shows how the tech bosses celebrate and praise Trump. The video not just shows the interconnection of big capital and big power, which exists in all forms and phases of capitalism, but it also shows that Silicon Valley’s digital capital elite favours Trump because they expect him to entirely deregulate the digital economy in and beyond the USA so that digital capital can globally do whatever it pleases in order to accumulate every more capital. That is why they celebrate Trump as an incredible pro-business and pro-innovation leader who unleashes digital innovation and creativity, etc. The unsettling reality is that the video shows that (digital) capital does not care about democracy and might support democracy today and authoritarianism tomorrow, depending on what system enables better capital accumulation.

Imitation has been one of the EU’s attempt to counter digital monopolies and their ideological logic. Imitation means that public funding is used for trying to stimulate the creation of competing digital giants (big tech). An example was the European Union’s Lisbon Strategy, which formulated the goal that the EU by 2010 wanted to become the world’s leading information economy that was able to catch up with and outcompete Silicon Valley’s digital giants (big tech). The strategy did not work out. No European digital giants were created. Rather, China created several imitations of Silicon Valley platforms, such as TikTok, that, just like the Silicon Valley platforms, are based on the logic of advertising and tabloidisation and, in addition, on strong state control of online information and communication. Basing digital democracy’s information, communication, and co-production processes on Silicon Valley’s digital capitalist platforms and China’s digital state monopoly capitalist platforms can undermine digital democracy’s resilience. It can make democratic information and communication prone to political and ideological disruption strategies, including fake news, surveillance, and censorship. Therefore, making digital democracy resilient requires other strategies than imitation.

The regulation of big tech and the digital giants is another strategy. For example, the European Union has passed a number of legal acts that question whether global digital corporations should be able to do anything that they want to do, especially with respect to market concentration, privacy, and data protection. Relevant policies include, for example, the General Data Protection Regulation (GDPR), the Digital Services Act (DSA), the Digital Markets Act (DMA), the AI Act, the Cyber Resilience Act (CRA), the Transparency and Targeting of Political Advertising Regulation, the Regulation on Open Internet Access, the Regulation on Geo-blocking, and the Data Act. Laws that advance privacy, data protection, anti-trust, etc., are certainly important. However, if there are monopolies and oligopolies controlled by companies located in a single country, legislation made in other countries or regions, such as the EU, can be ignored or circumvented by no longer offering such platforms in countries that regulate their use. While legislation and regulation are predominantly national and regional, the digital giants operate globally. The contradiction between the global digital economy and national/regional legislation poses limits on what can be achieved by regulating the digital giants. As a consequence, operating digital democracy’s information, communication, and co-production processes on the digital giants’ platforms risks economic and cultural disruption. Making digital democracy resilient certainly requires democratic regulation. Regulation alone is, however, not sufficient. Resilient digital democracy requires democratic autonomy from the digital giants, which in turn requires democratic alternatives.

The political theorist David Chandler argues that resilience thinking in society and the social sciences challenges “top-down ‘elitist’ assumptions of management and control from above” and supports the idea of autonomy as “spontaneous or autonomous self-activity” so that humans realise themselves “as the ‘real’ power at work in producing wealth and reproducing the world” ( Chandler, 2014, 59). In the context of digital platforms and digital democracy, this means that for digital democracy to work in a resilient manner, its infrastructures should be made autonomous from corporate control, state control, and ideological control. Chandler, in this context, refers to theories of autonomy, such as the one by Antonio Negri, who bases his thinking on Spinoza.

For Spinoza, potestas (Power) is dictatorial Power ( *dictoria potestas*): “So since dictatorial power [dictoria potestas] is absolute, it is bound to be a terror to all, especially if, as is here required, there is a fixed time for a dictator to be appointed” ( Spinoza, 1675–1677/2002, 747–748). Opposing dictatorship, he speaks of the multitude’s power ( *potentia*) as “the power of a people” ( *potentia multitudinis)* that “is usually called sovereignty” ( Spinoza, 1675–1677/2002, 687). Negri builds on Spinoza’s differentiation between potestas (top-down-Power) and potentia (power-from-below) for arguing that Spinoza sees “the best constitution [ … ] founded on [ … ] the affirmation of autonomous forces” ( Negri, 1991, 202). Negri argues that potentia and potestas mean “human power versus absolute Power” ( Negri, 1991, 69). From power as potential that challenges absolute Power, Negri derives his notion of autonomy. “By way of an autonomous dynamic, the human *conditio* becomes political *constitutio*; […] The autonomy of the political can only be constituted by the autonomy of a collective subject. […] only power, by constituting itself, only the power of the many, by making itself collective constitution, can found a Power. In this framework, Power is not seen as a substance, but rather as the product of a process aimed at collective constitution, a process that is always reopened by the power of the multitude” ( Negri, 2004, 15). In the context of digital political economy, digital potestas means that a group controls digital media by economic, political, or ideological means in order to exert its influence against the will of other groups.

Digital potestas signifies the undemocratic control of digital media. In contrast, digital potentia means self-managed digital media that try to serve the common interest so that all benefit. Digital autonomy and autonomisation form a strategy of digital democratic resilience where digital potestas is challenged by creating spaces of digital potentia, digital democratic spaces. “Not-for-profit digital projects that take on forms such as Creative Commons, platform co-operatives, public service Internet platforms, digital commons, etc. have the digital potentia to challenge digital capital’s Power” ( Fuchs, 2025a, 22). The basic idea of digital autonomy as resilience is that digital democracy is more persistent when particularistic interests by small groups are avoided, and that this can best be enabled by platforms that are autonomous from the power of private individuals’ money, political dictatorship, and ideological bias. Ideological bias of platforms means that certain ideas that distort reality are dominate, privileged, or especially supported by Internet platforms, their algorithms, owners, etc.

There are parallels between Negri’s notion of autonomy and Jürgen Habermas’ concept of the democratic public sphere. A public sphere is a publicly accessible space where information and communication about society takes place and public opinions are formed. In his *Structural Transformation of the Public Sphere* (first published in German in 1962), Habermas speaks of the logic of money and power as “refeudalisation” processes of the public sphere ( Habermas, 1989, 142, 158, 195, 200, 231). In his later work *Theory of Communicative Action*, he uses the notion of the “colonisation of the life-world” that includes the two processes of “monetarization and bureaucratization” ( Habermas, 1987, 321, 323, 325, 386, 403). According to Habermas (1987, 323, 343), monetarisation and bureaucratisation, the power of money and administration, instrumentalise and reify the lifeworld and thus the public sphere. What he thereby means is that the dominance of the logics of money and administrative power can destroy democracy. We can add a third colonising mechanism, namely ideology.

If reality is presented in distorted manners in order for trying to advance certain interests, we can speak of ideology. Ideology, as for example present in the form of fake news, undermines democracy. It is, besides commodification and domination, an important form of the colonisation of the public sphere ( Fuchs, 2024, 262). In the context of digital democracy, colonisation means that there are mechanisms that undermine citizens‘autonomous decision-making. These mechanisms are private platform ownership (digital capital, the digital giants, and digital monopolies that use strategies such as big data collection, micro-targeting, algorithmic analysis for hijacking and managing users’ attention, see Fuchs, 2024), state control of digital politics (digital censorship, repression, surveillance), and digital ideology (ideology is spread on digital networks). Autonomy means that digital democracy is resilient against economic, political, and ideological control. Establishing and operating an autonomous democratic platform are measures that try to make democracy resilient against colonisation and feudalisation.

The European Union’s digital policy making has, with respect to the Internet, thus far followed *two phases*: *imitation (1990s, 2000s) and regulation (since 2010*). The EU faces the danger that in the future, the digital giants will, backed by the governments of the US and China, simply ignore EU legislation and exclude EU users from accessing these platforms. The consequence would be that Internet use in the EU would severely decline as it depends on US and Chinese platforms. In addition to the questioning of EU Internet policies, the EU’s Internet also faces a high level of misinformation and foreign interference in politics and election campaigns in the form of fake news, hacking, hybrid warfare, deep fakes, post-truth, online hatred, and algorithmic politics. Given that the EU’s critical Internet infrastructure is dependent on the US and Chinese global digital giants and faces attacks, it is not resilient. Therefore, we see the need for the EU to enter a new stage of Internet policy making, the phase of Internet and digital autonomy and sovereignty. Internet and digital autonomy mean the advancement of policies that create and strengthen an autonomous EU Internet infrastructure, including major Internet platforms, that operates from within the EU, adheres to democratic European principles and EU regulation. Autonomy is the best strategy to make the EU’s Internet resilient and defend digital democracy in the EU.

One important alternative for countering online fake news is fact-checkers. Fact checkers are news professionals who check the truth of news and report on specific news items. Supporting fact-checking legally, institutionally, and financially is an important measure for advancing resilient digital democracy. For example, higher education institutions can develop fact-checking courses. Fact-checkers require academic skills to check the correctness of news. For example, they need to be able to conduct searches for relevant academic works, properly read and interpret academic studies and statistical data, write critical reports, etc. Legislation can create laws that give fact-checking organisations an important role in the public sphere. Finally, sufficient public and private funding is required for making fact-checking work. Fact-checking organisations require adequate resources, including funding, fact-checking technologies, and highly educated and well-paid fact-checking professionals. They face the problem that the creation and dissemination of fake news and disinformation operates at high speed and is partly or fully automated while fact-checking requires significant amounts of labour-time. Therefore, the issue arises of how to best manage the antagonism between the high speed of fake news and the slow speed of fact-checking. Fact-checking needs to be combined with other measures such as the creation of alternative, non-commercial, democratic Internet platforms.

Platform co-operatives and public service Internet platforms are democratic alternatives to the digital giants that have the potential to support the creation of an autonomous and sovereign EU Internet. Platform co-operatives are Internet platforms that are controlled and managed by their workers and users. A platform co-operative is “a project or business that primarily uses a website, mobile app, or protocol […] and relies on democratic decision-making and shared community ownership of the platform by workers and users” ( Scholz, 2023, 8). The idea of platform co-operatives comes out of the co-operative movement.

Public service media are media that are enabled by special legislation, do not operate for-profit, are to a large degree non-commercial, are funded by the public (often in the form of a licence fee), and have a public service media remit that includes the legally defined mandate to inform, provide high quality impartial news and educational programmes, entertain, and promote democracy and democratic information and communication. Public service media are only truly public if they are independent from corporate, state, and ideological power. Public service Internet platforms are Internet platforms run by public service media. “The digital giants have weakened democracy and the Internet. We need a new Internet. We need to rebuild the Internet. While the contemporary Internet is dominated by monopolies and commerce, the Public Service Internet is dominated by democracy. While the contemporary Internet is dominated by surveillance, the Public Service Internet is privacy friendly and transparent. While the contemporary Internet misinforms and separates the public, the Public Service Internet engages, informs and supports the public. Although the contemporary Internet is driven by and drives the profit principle, the Public Service Internet puts social needs first” ( PSMI Manifesto Collective, 2021, 13).

The advantage of platform co-operatives and public service Internet platforms is that they are not privately owned and, therefore, do not give single individuals huge communication power in the public sphere. Both models are, therefore, suited as alternative organisational models for digital democracy platforms. For example, a specific digital democracy project can be operated and controlled by a local council or organised as a platform co-operative organised by the citizens in the local community. Digital democracy software is, to a significant degree, free, libre and open source software that can be used, adapted, and further developed without large costs. Examples are the digital democracy platforms Decidim ( https://decidim.org/), Consul ( https://consuldemocracy.org/), and pol.is ( https://pol.is/home). Local digital democracy projects can, without much effort, use such software. Doing so enables them to operate independently from the digital giants. Using free, libre and open source (FLOSS) software is therefore another important strategy for advancing resilient digital democracy. Overall, using, developing, and supporting platform co-operatives, public service Internet platforms, as well as free, libre and open source (FLOSS) software in the context of digital democracy is an important economic and cultural strategy for making digital democracy resilient.

The weakness of alternative, non-commercial, democratic Internet platforms is that they often remain small, unknown, and resource-poor. Wikipedia is one of a few not-for-profit exceptions that have gained public attention. Others include, for example, OpenStreetMap (run by the OpenStreetMap Foundation), Creative Commons, Wayback Machine (run by the Internet Archive), or FLOSS (free/libre/open source software). Organisations such as Wikimedia or other not-for-profit civil society tech organisations could play an important role in making digital democracy resilient. One problem of public service media organisations is that they are often bureaucratic, which means they may lack the flexibility and dynamics needed to develop digital technologies, and are prone to political and state control. State-controlled public service media are no longer public service but rather state media.

Overcoming such weaknesses might be achieved by networking and combining public service Internet projects, platform co-operatives, and civil society Internet projects. Instead of private/public partnerships where public administration and public organisations partner with for-profit tech corporations so that taxpayers’ money is turned into private profits of digital tech corporations that provide tech solutions. Replacing public/private partnerships with public/commons partnerships that network public administration, public organisations (including public universities, libraries, museums, theatres, etc.), platform co-operatives, and civil society organisations in the provision of digital democracy platforms. “Public Service Internet platforms maximise the availability and permanence of Public Service Internet contents that contribute to humanity’s cultural heritage. Public Service Internet platforms are ideally operated as international networks of Public Service Media organisations. For operating Public Service Internet platforms, Public Service Media organisations co-operate with others, including public organisations (universities, museums, libraries and so on), civil society, civic and community media, artists, digital commons projects, platform co-operatives, and so on. There is a sharing of content between such public and civic organisations on a joint platform. As a result, Public Service Media organisations together with public interest organisations create public open spaces that are mediated by Internet communication and that together form the Public Service Internet” ( PSMI Manifesto Collective, 2021, 15).

The idea of combining platform co-operatives and public service Internet platforms in digital public/commons partnerships is an update of the cultural theorist Raymond Williams’ suggestion to create a “cultural democracy” ( 1973, 121) that combines public-service media, cultural co-operatives and local media and where “public ownership of the basic means of production [the means of communication and cultural production] should be combined with leasing their use to self-managing groups, to secure maximum variety of style and political opinion and to ensure against any bureaucratic control” ( Williams 1979, 370).

Platform co-operatives, civil society organisations, and public organisations are not automatically dedicated to (digital) democracy. They can also represent authoritarian or fascist interests that question, undermine, and try to destroy democracy. In such cases, they are not committed to the public and the common good. Authoritarianism means the dominance and rule of single persons or groups over the rest of the population. In contrast, democracy is a common form of politics where power is exerted collectively by all from the bottom-up, which, depending on the model of democracy, takes on different forms such as representation, participation, deliberation, the combination of representation and participation, etc. Public/common partnerships are not immune to authoritarianism. They are, however, more likely to represent the public and common interest than privately owned corporations that politically often represent the interests of individuals who own the company. Examples are Rupert Murdoch’s news media, such as Fox News, that are politically reporting in a conservative manner, and X, where Elon Musk, who owns the platform, has huge communication power and is the user with the highest number of followers and the highest level of visibility. Public and common organisations do not amass economic and political power in the hands of single individuals. They tend to be democratically governed. This experience and practice of democracy in the organisation itself makes them more likely than private corporations to represent democratic ideals such as the commitment to basic and human rights and democracy as such.

However, there can also be what Amlinger & Nachtwey (2025) term “democratic fascism”, where democracy is used to advance fascism, which does not mean that fascism is democratic, but that fascism uses the structures, concepts, rhetoric, and language of democracy to present itself as democratic and democrats as undemocratic in order to successively undermine democracy from within. Lührmann and Lindberg (2019) identified 217 instances of autocratisation in a total of 109 countries in the years from 1900 until 2017. They speak of three waves of autocratisation (1926-1942, 1961-1977, since 1994). The third wave corresponds to what Amlinger and Nachtwey describe as democratic fascism, fascism’s hijacking of democracy: “Democratic erosion became the modal tactic during the third wave of autocratization. Here, incumbents legally access power and then gradually, but substantially, undermine democratic norms without abolishing key democratic institutions. Such processes account for 70% in the third reversal wave with prominent cases of such gradual deterioration” ( Lührmann & Lindberg, 2019, 1105). The implication of such developments is that public and commons projects need a deliberate commitment to democracy. The implication is that public and commons projects need a deliberate commitment to democracy.

## 6. Conclusion

This paper developed a comprehensive theoretical foundation for understanding resilient digital democracy in an era marked by polarisation, accelerating crises, and the rise of digital authoritarianism. It traced the concept of resilience from systems theory, where it describes the capacity to absorb shocks without collapse, to social and political contexts, emphasising that democratic resilience is not merely a property of systems but a human-driven practice of resistance, renewal, and collective agency. Democracy’s vulnerability becomes acute when environmental, economic, technological, political, and cultural crises interact, enabling authoritarian movements to exploit disinformation, surveillance, digital monopolies, and the erosion of trust.

The author distinguishes between democratic resilience (democracy as an attribute of resilience itself ) and resilient democracy (resilience as a feature of democracy), arguing for the latter as a more accurate framing. Resilient democracy involves both subjective commitment to democratic ideals and objective structures that prevent democratic backsliding. These dynamics unfold across two temporal stages: onset resilience, which halts the early emergence of authoritarianism, and breakdown resilience, which protects democracy at advanced stages of erosion.

The paper then showed that digital democracy, which encompasses, for example, participatory budgeting, online assemblies, civic platforms, and cyber activism, is itself exposed to multiple forms of disruption, from natural disasters and cyberattacks to platform monopolies, censorship, and the cultural spread of fake news and hate speech. To strengthen digital democracy, the author proposed an integrated set of strategies: decentralised data storage, advanced security infrastructures, democratic regulation of digital giants, strong constitutional protections, and the revitalisation of democracy through participatory and deliberative tools. The paper particularly highlighted platform co-operatives, public service Internet platforms, and free/open-source digital democracy infrastructures as alternatives to privately controlled platforms that concentrate communicative power. Such forms of organising the digital are forms of digital potentia, important infrastructures of digital democracy that make the latter resilient, that is, autonomous from digital capital, digital state control, and digital ideology. Ultimately, resilient digital democracy requires hybrid forms of engagement that blend the trust-building capacity of offline interaction with the accessibility and scale afforded by digital tools.

Building resilient digital democracy in the years ahead will require deliberate political will, institutional innovation, and societal commitment to democratic values. As authoritarian actors increasingly exploit digital infrastructures, resilience must be cultivated through public-interest models of digital technologies, robust civic education, and democratic governance models that empower citizens rather than data-driven monopolies. Strengthening the democratic quality of digital spaces through participatory platforms, transparent algorithms, secure infrastructures, and public commons partnerships will be essential for navigating future crises. The challenge is not merely to protect democracy from digital threats, but to harness digital technologies to deepen inclusion, trust, and collective problem-solving. If societies can align democratic principles with technological design, digital democracy can become not only more resilient but a driver of democratic renewal.

## Ethics and consent

Ethical approval and consent were not required.

## Data Availability

No data was collected for this article.

## Footnotes

^1^
Search conducted on 14 November 2025 in: https://scopus.com, https://webofscience.com
