# Supplementary material for: What is and how do we achieve a resilient digital democracy?
Source: Open Res Eur. 2026 Feb 10;5:387. Originally published 2025 Dec 12. [Version 2] doi: 10.12688/openreseurope.21988.2 (PMC12869019; doi:10.12688/openreseurope.21988.2)
Supplement: Peer review history

# Reviewer report: Reviewer response for version 2

**DOI:** 10.21956/openreseurope.24931.r69391

**Published:** 2026-02-11

**Authors:** Michele Sorice

**Affiliations:** 1 Sapienza University of Rome, Rome, Italy

The new version is even better than the first one (that was, in my opinion, already excellent)

Is the argument persuasive and supported by appropriate evidence?

Yes

Is the work clearly and cogently presented?

Yes

Is the topic of the essay discussed accurately in the context of the current literature?

Yes

Does the essay contribute to the cultural, historical, social understanding of the field?

Yes

Reviewer Expertise:

Media and Democracy, Digital Media, Digital Democracy, Citizenship, Social movements and media

I confirm that I have read this submission and believe that I have an appropriate level of expertise to confirm that it is of an acceptable scientific standard.

# Reviewer report: Reviewer response for version 1

**DOI:** 10.21956/openreseurope.23791.r68453

**Published:** 2026-02-03

**Authors:** Michele Sorice

**Affiliations:** 1 Sapienza University of Rome, Rome, Italy

This article makes an exceptionally relevant and timely theoretical contribution to the contemporary debate on digital democracy and its ability to withstand authoritarian challenges. The author presents an innovative and robust conceptual framework that successfully combines systems theory, critical sociology, digital political economy and democratic theory.

 The article articulates a critical genealogy of the concept of resilience, tracing its evolution from systems theory (Holling) to socio-political applications and moving beyond the structuralist–functionalist approach that dominates existing literature. Privileging a perspective centred on human agency over systemic properties is a significant epistemological contribution that restores centrality to collective political action.

 The systematisation of literature on resilient democracy in section 3 is impressive in its completeness and analytical rigour. Rather than merely cataloguing existing definitions, the author critically identifies their limitations and proposes an original synthesis that integrates subjective and objective dimensions, structures, and practices. The conceptual distinction between 'democratic resilience' and 'resilient democracy' is particularly valuable, as it clarifies terminological ambiguities in the literature.

 From a media studies perspective, the article provides an in-depth analysis of threats to digital democracy, ranging from technological monopolies and fake news to algorithmic surveillance and digital acceleration. It goes beyond superficial descriptions to identify the underlying structural logics of commercial colonisation, state control and the ideological distortion of the digital public sphere.

 The proposal of multidimensional strategies for resilient digital democracy (Table 3) constitutes the work's most innovative core. Integrating environmental, technological, political and economic-cultural dimensions demonstrates a sophisticated understanding of the complexity of digital democratic infrastructures. 

 The argument in favour of cooperative platforms, digital public service media, and free software as infrastructures of resilience is particularly convincing. The author avoids naive techno-optimism, lucidly recognising the limits and contradictions of these models (e.g. tendency to remain marginal, risks of bureaucratisation, possible authoritarian co-optation), and proposing concrete solutions such as public-commons partnerships that transcend public-private logic.

 The critical perspective on the European Union is illuminating. The periodisation of EU digital policies identifies a precise historical trajectory and proposes an alternative centred on European democratic digital sovereignty.

 In summary, this is an exceptionally mature work of theory that significantly advances the state of the art in resilient digital democracy, offering refined conceptual tools and concrete strategic guidance.

Is the argument persuasive and supported by appropriate evidence?

Yes

Is the work clearly and cogently presented?

Yes

Is the topic of the essay discussed accurately in the context of the current literature?

Yes

Does the essay contribute to the cultural, historical, social understanding of the field?

Yes

Reviewer Expertise:

Media and Democracy, Digital Media, Digital Democracy, Citizenship, Social movements and media

I confirm that I have read this submission and believe that I have an appropriate level of expertise to confirm that it is of an acceptable scientific standard.

# Reviewer report: Reviewer response for version 1

**DOI:** 10.21956/openreseurope.23791.r68458

**Published:** 2026-01-29

**Authors:** Reginaldo Gonçalves Gomes

**Affiliations:** 1 PHILOSOPHY, FEDERAL UNIVERSITY OF OURO PRETO, Belo Horizonte, MINAS GERAIS, Brazil

**REPORT**

**Review of “What is and how do we achieve a resilient digital democracy?”** **By Christian Fuchs**

**Major issues:**

****The article makes an important contribution and is suitable for indexing; however, some revisions should be addressed prior to indexing.

             The aim of this article is to address the following questions: What is a resilient digital democracy? How can such a form of digital democracy be achieved? These questions necessarily involve the concept of democracy itself, not only the notion of resilience, as well as the digital dimension, which implies that authors who write on information and communication technologies should be engaged.

             In introduction, the author affirms, “The world is today politically highly polarised. Multiple crises have cascaded into a negative dialectic where global neoliberal capitalism has turned against liberal democracy, which has resulted in the emergence and success of new authoritarian movements.”

             Limiting the debate about resilient democracy and digital democracy to global neoliberal capitalism versus liberal democracy ignores the broader political discussion between left-wing and right-wing ideologies, such as socialism and liberalism.

             In the world, for example, in Latin America, the debate involves the left wing and the right wing. The left wing is identified as being socialist and the right wing as being liberal, and there are also extreme right-wingers.

             The author discusses digital democracy, but the article does not engage with authors who specifically address information and communication technologies. In addition, the article lacks references to political philosophers who are central to the concept of democracy.

 The author could have cited philosophers who write about consciousness, morality, and norms. By citing only sociologists, the analysis fails to capture the full dimension of the human being as a self-conscious agent endowed with consciousness and unconsciousness, reason, and reflection. As a result, the article presents a conception of the human being that is exclusively social.

**This is only a suggestion for the author and does not imply rejection of the article if it is not accepted.**

**Minor issues:**

**In the following, I offer some more specific comments.**

**On page 3:** Holling and Hofkircher are cited, but the author did not mention the year of the book, for he made a paraphrase.

**The author should indicate the year of publication of the works cited.**

**On page 4:** “The system boundary the delaminates[1] the system from its environment and the system’s dynamic (the behaviour that the elements in relation to each other show as reaction to inputs) continue to exist.”

             It is better to use the verb “to delimit” or “limit”. There is also a mistake in using the article “the” before “delaminates”; it should be “that”.

 The author should correct the phrase as follows: “The system boundary **that** **limits **(or delimits)...”

**On page 9: **the author affirms, “Political disruption means that digital democracy platforms are censored, monitored, outlawed, or that violence is used against digital democracy operators or users.”

 The sentence suggests that the government cannot restrict digital platforms when they are committing crimes?

             A **democratic government is fully justified** in imposing **fines, platform bans, or imprisonment** when digital platforms are used to commit **serious crimes**, including the **production, distribution, or display of illegal content involving children** **.**

**The author should clarify this definition.**

**On page 10**: *“[...] Digital democracy is economically disrupted when platforms that advance and support authoritarianism dominate the public sphere, or there is a lack of funding. The online spreading of fake news, deep fakes, hate speech, disinformation and post-truth culture, the formation of online echo chambers, and digital tabloidisation..[...]”.*

 It feels like there are human beings behind those acts; the author did not name who really would like to threaten democracy. In the beginning, he asserts that “where global neoliberal capitalism has turned against liberal democracy, which has resulted in the emergence and success of new authoritarian movements.”

  But that threat to democracy only comes from neoliberal capitalism? What about the extreme liberals who share most of the same ideology as the liberal one? What about the extreme left who share the same ideology as his mates who are not extreme ones?

 We should not overlook the fact that neoliberalism is an ideology that emerged from classical liberalism.

 In my opinion, it is not “the neoliberals,” “the authoritarians,” “the far-left,” or “the liberals” as monolithic blocks that spread disinformation. Instead, specific actors within these spectrums, especially those with authoritarian, populist, or radical tendencies, adopt disinformation as a strategic tool. The current digital ecosystem, shaped by algorithms that reward extreme engagement, enables these tactics to spread rapidly, regardless of ideological origin.

**So, I would like the author to clarify this sentence.**

**On page 10:** “Authoritarianism and digital fascism”.

 I think there is no polarisation between neoliberal capitalism and liberal democracy, for neoliberal capitalism is just a vert of liberal democracy.

 So, who are the authoritarians and fascists? Neoliberal capitalists? Do neoliberal capitalists have sides or just want to make more money? So, I think the polarisation is between left and right or socialists and liberals. And among them, there are authoritarians and fascists.

**So, I would like the author to clarify in the text who the authoritarians and fascists are.**

**On page 11:** “The basic idea of digital autonomy [...] and that this can best be enabled by platforms that are autonomous from the power of private individuals’ money, political dictatorship, and ideological bias.

             When discussing ideological bias, we are referring to a type of cognitive bias. So, what kind of cognitive bias is it? **The text needs a footnote to explain which cognitive bias this is.**

**On page 12:** “Authoritarianism means the dominance and rule of single persons or groups over the rest of the population.”

 Hannah Arendt, for example, defines authoritarianism as a regime in which power is concentrated in a single authority, whether an individual, a party, or the military, that does not tolerate opposition while allowing relatively autonomous spheres of private life. What is decisive is the suppression of political freedom and plurality, not the total penetration of private life characteristic of totalitarian regimes.

**The concept of authoritarianism used here is not referenced in the text. Which author defines it as the domination exercised by a group or individual? Or is this the author’s own definition?**

**Page 13:** “However, there can also be democratic forms of fascism (Amlinger & Nachtwey, 2025) where democracy is used to advance fascism.”

 The phrase “democratic forms of fascismo” is a contradiction, as fascism is fundamentally anti-democratic. The standard definition of fascism is a political ideology and regime characterized by extreme nationalism, mass mobilization, anti-liberalism, anti-pluralism, and the concentration of power in an authoritarian leader.

**I suggest the author revise this statement.**

**Final recommendation**

 Competing Interests: No competing interests were disclosed.

 I confirm that I have read this submission and believe that I have an appropriate level of expertise to assess its scientific standard; however, I have some revisions to suggest to the author, as outlined above.

**[1]** It’s a technical term used in **materials science, engineering, geology, and medicine** **.**

Is the argument persuasive and supported by appropriate evidence?

Partly

Is the work clearly and cogently presented?

Yes

Is the topic of the essay discussed accurately in the context of the current literature?

Yes

Does the essay contribute to the cultural, historical, social understanding of the field?

Yes

Reviewer Expertise:

philosophy of mind, ethics,

I confirm that I have read this submission and believe that I have an appropriate level of expertise to confirm that it is of an acceptable scientific standard, however I have significant reservations, as outlined above.

# Response

**Published:** 2026-01-30

**Authors:** Christian Fuchs

**Response to review 2, by Christian Fuchs (author)** **Author:**

 Thank you for the constructive review, which has helped me to improve the article. I created a new version that built in your review comments and a second review comment.

**Reviewer:** The article makes an important contribution and is suitable for indexing; however, some revisions should be addressed prior to indexing. **Reviewer:** The aim of this article is to address the following questions: What is a resilient digital democracy? How can such a form of digital democracy be achieved? These questions necessarily involve the concept of democracy itself, not only the notion of resilience, as well as the digital dimension, which implies that authors who write on information and communication technologies should be engaged.

**Author:**

 It is true that this also concerns ICTs and studies of fit. In this paper, I have a strong focus on digital resilience and resilient digital democracy, not digital democracy in general, so I have focused the referenced literature on authors who write about digital resilience and resilient digital democracy, which are not so many. I have added a reference to and quote from a research report from the EU project this paper comes out of where I and my research team have thoroughly engaged with the notion of digital democracy (Fuchs 2025b)…

 =>

 “Digital democracy means the mediation of democracy by digital information and communication technologies. “Digital democracy is the use of digital media in the practice of democracy. It refers to the digital mediation of democracy. Digital democracy is the mediation of democracy by a variety of digital media, including, but not limited to, the Internet, the WWW, smartphones, apps, tablets, social media, online discussion forums, electronic voting systems, computer-supported co-operative work technologies, artificial intelligence technologies, audio and video streaming platforms, podcasts, etc. […] The features and affordances of digital technology use, such as convergence and presumption (productive consumption), shape the practices and models of digital democracy. Digital democracy has three encapsulated symbolic dimensions: the production and sharing of democratic information (digital democratic information), democratic communication (digital democratic communication), and the co-creation of democracy (digital democratic co-production)“ (Fuchs 2025b, 88-89). Reviewing the state of the art of digital democracy theory and research, Fuchs (2025b) identifies six models of digital democracy: constitutional digital democracy, deliberative digital democracy, participatory digital democracy, representative digital democracy, direct digital democracy, and pluralist digital democracy.”

**Reviewer:** In introduction, the author affirms, “The world is today politically highly polarised. Multiple crises have cascaded into a negative dialectic where global neoliberal capitalism has turned against liberal democracy, which has resulted in the emergence and success of new authoritarian movements.”

 Limiting the debate about resilient democracy and digital democracy to global neoliberal capitalism versus liberal democracy ignores the broader political discussion between left-wing and right-wing ideologies, such as socialism and liberalism. In the world, for example, in Latin America, the debate involves the left wing and the right wing. The left wing is identified as being socialist and the right wing as being liberal, and there are also extreme right-wingers.

**Author:**

 I have a broad understanding of capitalism: it is a global world system that not just includes the economy, but also politics, culture, etc. In my approach, there is a capitalist world system so the problems are within this system. I have further outlined this issues, also referring to more philosophical writings, namely to Horkheimer and Adorno who are important to my approach…

 I added:

 “Today, the world is politically highly polarised. Multiple crises have cascaded into a negative dialectic where global neoliberal capitalism has turned against liberal democracy, which has resulted in the emergence and success of new authoritarian movements. My analysis is here based on the two Frankfurt School philosophers Horkheimer and Adorno (2002), who argue in their book Dialectic of Enlightenment that there has been “the self-destruction of enlightenment“, the “enlightenment’s relapse into mythology“ (xvi), and that the enlightenment has the tendency of “the reversion of enlightened civilization to barbarism in reality“ (xix). They argue that the negative dialectic of enlightenment resulted in Auschwitz, world wars, fascism, and authoritarianism. According to Horkheimer and Adorno, the cause of the negative dialectic of the Enlightenment is that reason was turned into a means of domination and control. Humans are reified and alienated, which means that they become instruments that serve purposes that lie outside of their own interests. Today, we live in a capitalist world system where the economic, environmental, social, political and ideological antagonisms have cascaded into multiple crises that have advanced new forms of authoritarianism, fascism, and nationalism.”

**Reviewer:**

 The author discusses digital democracy, but the article does not engage with authors who specifically address information and communication technologies. In addition, the article lacks references to political philosophers who are central to the concept of democracy.

 The author could have cited philosophers who write about consciousness, morality, and norms. By citing only sociologists, the analysis fails to capture the full dimension of the human being as a self-conscious agent endowed with consciousness and unconsciousness, reason, and reflection. As a result, the article presents a conception of the human being that is exclusively social.

**Author:**

 I consider philosophy very important, so your point is a good one. I have added a discussion of aspects outlined by Fromm, Horkheimer, Adorno, Arendt, Habermas, and Dreyfus to my argumentation, which gives the paper a bit of a philosophical twist.

**Reviewer:**

 On page 3: Holling and Hofkircher are cited, but the author did not mention the year of the book, for he made a paraphrase.

 The author should indicate the year of publication of the works cited. **Author:** I added the publication years

**Reviewer:**

 On page 4: “The system boundary the delaminates[1] the system from its environment and the system’s dynamic (the behaviour that the elements in relation to each other show as reaction to inputs) continue to exist.”

 It is better to use the verb “to delimit” or “limit”. There is also a mistake in using the article “the” before “delaminates”; it should be “that”.

 The author should correct the phrase as follows: “The system boundary that limits (or delimits)...”

**Author:**

 Done.

**Reviewer:**

 On page 9: the author affirms, “Political disruption means that digital democracy platforms are censored, monitored, outlawed, or that violence is used against digital democracy operators or users.”

 The sentence suggests that the government cannot restrict digital platforms when they are committing crimes?

 A democratic government is fully justified in imposing fines, platform bans, or imprisonment when digital platforms are used to commit serious crimes, including the production, distribution, or display of illegal content involving children.

 The author should clarify this definition.

**Author:**

 No, that sentence has a different meaning, I speak of the censorship, outlawing of DEMOCRATIC platforms, if they promote violence, terror, etc., then Internet platforms are not democratic, so the imlicaiton is they can and should be censored.

**Reviewer:**

 On page 10: “[...] Digital democracy is economically disrupted when platforms that advance and support authoritarianism dominate the public sphere, or there is a lack of funding. The online spreading of fake news, deep fakes, hate speech, disinformation and post-truth culture, the formation of online echo chambers, and digital tabloidisation..[...]”.

 It feels like there are human beings behind those acts; the author did not name who really would like to threaten democracy. In the beginning, he asserts that “where global neoliberal capitalism has turned against liberal democracy, which has resulted in the emergence and success of new authoritarian movements.”

 But that threat to democracy only comes from neoliberal capitalism? What about the extreme liberals who share most of the same ideology as the liberal one? What about the extreme left who share the same ideology as his mates who are not extreme ones?

 We should not overlook the fact that neoliberalism is an ideology that emerged from classical liberalism.

 In my opinion, it is not “the neoliberals,” “the authoritarians,” “the far-left,” or “the liberals” as monolithic blocks that spread disinformation. Instead, specific actors within these spectrums, especially those with authoritarian, populist, or radical tendencies, adopt disinformation as a strategic tool. The current digital ecosystem, shaped by algorithms that reward extreme engagement, enables these tactics to spread rapidly, regardless of ideological origin.

 So, I would like the author to clarify this sentence.

 On page 10: “Authoritarianism and digital fascism”.

 I think there is no polarisation between neoliberal capitalism and liberal democracy, for neoliberal capitalism is just a vert of liberal democracy.

 So, who are the authoritarians and fascists? Neoliberal capitalists? Do neoliberal capitalists have sides or just want to make more money? So, I think the polarisation is between left and right or socialists and liberals. And among them, there are authoritarians and fascists.

 So, I would like the author to clarify in the text who the authoritarians and fascists are. **Author:**

 For the clarification of the sentence “where global neoliberal capitalism has turned against liberal democracy, which has resulted in the emergence and success of new authoritarian movements.”, please see my comment above and my respective change of the paper with references to Horkheimer and Adorno

 In respect to the reviewer’s comment “Digital democracy is economically disrupted when platforms that advance and support authoritarianism dominate the public sphere, or there is a lack of funding. The online spreading of fake news, deep fakes, hate speech, disinformation and post-truth culture, the formation of online echo chambers, and digital tabloidisation..[...]”, I added the following paragraph in order to pinpoint the power of big tech:

 “An example of the intersection of economic, political and ideological power in global digital capitalism is that Silicon Valley big tech gurus such as Elon Musk, Peter Thiel, Marc Andreessen, Mark Zuckerberg, etc. have supported Donald Trump. After buying Twitter and turning it into X, Elon Musk got rid of fact-checking and the ban of extremist accounts. In January 2025 when Trump started his second presidential term, Zuckerberg ended Meta’s fact-checking programme. He characterised fact-checking as “too politically biased“ (Zuckerberg 2025). Fact-checkers are professional journalists who know how to check if certain claims are true or false. They are part of a news culture that works for free expression and against political bias. Critics argued that the decision effectively benefits far-right disinformation ecosystems by reducing moderation capacity. On 4 September 2025, Donald Trump hosted a dinner with top Silicon Valley big tech CEOs at the White House. They included Sam Altman (OpenAI: ChatGPT), Tim Cook (Apple), Bill Gates (Microsoft), Safra Katz (Oracle), Sundar Pichai (Alphabet: Google, YouTube), and Mark Zuckerberg (Meta: Facebook, Instagram, WhatsApp). An iconic video ( https://www.youtube.com/watch?v=dsl_sKYywEI, accessed on 30 January 2025) shows how the tech bosses celebrate and praise Trump. The video not just shows the interconnection of big capital and big power, which exists in all forms and phases of capitalism, but it also shows that Silicon Valley’s digital capital elite favours Trump because they expect him to entirely deregulate the digital economy in and beyond the USA so that digital capital can globally do whatever it pleases in order to accumulate every more capital. That is why they celebrate Trump as an incredible pro-business and pro-innovation leader who unleashes digital innovation and creativity, etc. The unsettling reality is that the video shows that (digital) capital does not care about democracy and might support democracy today and authoritarianism tomorrow, depending on what system enables better capital accumulation. “

**Reviewer:** On page 11: “The basic idea of digital autonomy [...] and that this can best be enabled by platforms that are autonomous from the power of private individuals’ money, political dictatorship, and ideological bias. When discussing ideological bias, we are referring to a type of cognitive bias. So, what kind of cognitive bias is it? The text needs a footnote to explain which cognitive bias this is.

**Author:**

 I added a sentence that clarifies what I mean by “ideological bias”

**Reviewer:**

 On page 12: “Authoritarianism means the dominance and rule of single persons or groups over the rest of the population.”

 Hannah Arendt, for example, defines authoritarianism as a regime in which power is concentrated in a single authority, whether an individual, a party, or the military, that does not tolerate opposition while allowing relatively autonomous spheres of private life. What is decisive is the suppression of political freedom and plurality, not the total penetration of private life characteristic of totalitarian regimes.

 The concept of authoritarianism used here is not referenced in the text. Which author defines it as the domination exercised by a group or individual? Or is this the author’s own definition?

**Author:**

 It’s a good point that the notion of authoritarianism should be better explained. I now did so. The main reference in my works on this topic is Frankurt School critical theory and its concept of authoritarianism. I point out some details, including references. These are philosophers, so as suggested by the reviewer, I am going more into philosophy by engaging with the Frankurt School authors and their works, Arendt, and others:

 “Given we are experiencing new forms of authoritarianism such as digital authoritarianism, the question arises of how we understand authoritarianism. I follow the Frankfurt School-approach for conceptualising authoritarianism (including authors such as Wilhelm Reich, Erich Fromm, Theodor W. Adorno, Max Horkheimer, Herbert Marcuse, and Franz L. Neumann). To understand authoritarianism, Wilhelm Reich argued for an approach that integrates critical psychology, political economy, and ideology critique. In 1936, the Frankfurt School released its study on authority and family. The goal was to understand where and how fascism originated (Horkheimer 1936/1987). Fromm defined the authoritarian personality as an individual who “exercises aggression against the defenseless and sympathy with the powerful“ (Fromm 1936/2020, 24). Fromm characterised the authoritarian personality as necrophilic. He shows that Hitler was a necrophile and that the “necrophile demands annihilation” (Fromm 1973/1997, 406), that Stalin was a mental and physical sadist, and that both stand for human destructiveness that is characteristic for authoritarianism that is opposed to humanism. Authoritarianism includes top-down-leadership structures, nationalism, the friend/enemy-scheme, and repression/violence (Fuchs 2022a). Authoritarians define outsiders and scapegoats who are blamed for the ills of society and social systems and against whom they propagate the use of repressive or even violent means. The advantage of the notion of authoritarianism, as utilized by the Frankfurt School, is that it is neither micro nor macro, but micro, meso, and macro: Authoritarianism and fascism are not organised at single levels, but take place at encapsulated levels where each level presupposes and goes beyond the underlying level: the individual, the group, social movements/parties, social systems/Institutions, and society (Fuchs 2024, 2022a).

 Fascism is a particular, radical form of authoritarianism. Its central feature is that it favours, propagates, uses, and institutionalises violence as the main political means and organisational principle of society. Violence is the fascists’ ultima ratio. The fascist “cannot help feeling surrounded by traitors, and so continuously threatens to exterminate them” (Adorno 1975, 78). In fascism, politics “grows out of the barrel of a gun” (Arendt 1970, 37), while in a democracy, politics grows out of the “generative force of communicative freedom” (Habermas 1996, 151). “Terror becomes total when it becomes independent of all opposition; it rules supreme when nobody any longer stands in ist way. If lawfulness is the essence of non-tyrannical government and lawlessness is the essence of tyranny, then terror is the essence of totalitarian domination“ (Arendt 1951/1973, 464).

 Arendt (1951/1973) draws a distinction between authoritarianism and totalitarianism: “authority, no matter in what form, always is meant to restrict or limit freedom, but never to abolish it. Totalitarian domination, however, aims at abolishing freedom, even at eliminating human spontaneity in general, and by no means at a restriction of freedom no matter how tyrannical” (405). While for Arendt the problem is totalitarianism,  for the Frankfurt School it is authoritarianism. Arendt sees totalitarianism as rooted in political breakdown (state, law, parties, citizenship). She gives a political explanation of it. In contrast, for the Frankfurt School authoritarianism arises from the administered world, a world that is ruled by instrumental reason. “

**Reviewer:**

 Page 13: “However, there can also be democratic forms of fascism (Amlinger & Nachtwey, 2025) where democracy is used to advance fascism.”

 The phrase “democratic forms of fascismo” is a contradiction, as fascism is fundamentally anti-democratic. The standard definition of fascism is a political ideology and regime characterized by extreme nationalism, mass mobilization, anti-liberalism, anti-pluralism, and the concentration of power in an authoritarian leader.

 I suggest the author revise this statement.

**Author:**

 I added a clarification:

 “However, there can also be what Amlinger and Nachtwey (2025) term “democratic fascism” where democracy is used to advance fascism, which does not mean that fascism is democratic, but that fascism uses the structures, concepts, rhetoric, and language of democracy to present itself as democratic and democrats as undemocratic in order to successively undermine democracy from within. Lührmann and Lindberg (2019) identified 217 instances of autocratisation in a total of 109 countries in the years from 1900 until 2017. They speak of three waves of autocratisation (1926-1942, 1961-1977, since 1994). The third wave corresponds to what Amlinger and Nachtwey describe as democratic fascism, fascism’s hijacking of democracy: “Democratic erosion became the modal tactic during the third wave of autocratization. Here, incumbents legally access power and then gradually, but substantially, undermine democratic norms without abolishing key democratic institutions. Such processes account for 70% in the third reversal wave with prominent cases of such gradual deterioration“ (Lührmann and Lindberg 2019, 1105).The implication of such developments is that public and commons projects need a deliberate commitment to democracy”

**Author:**

 Overall, the review has much helped me to improve the paper’s quality.

# Reviewer report: Reviewer response for version 1

**DOI:** 10.21956/openreseurope.23791.r66367

**Published:** 2026-01-12

**Authors:** Dirk Helbing

**Affiliations:** 1 ETH Zurich, Zürich, Zurich, Switzerland

**Review of**

**“What is and how do we achieve a resilient digital democracy?”**

**By Christian Fuchs**

 Overall, I have read the contribution with interest, and consider it worthy of indexing, but considering the comments below in a revision would further strengthen the manuscript. 

 The arguments provided add nicely to the recent discussion on digital democracy/ies.

 However, the literature appears to be unnecessarily restricted to political science references, while significant contributions have also been recently published in neighboring fields such as computational social science. It would be valuable to mention some of those activities as well. The same applies to participatory resilience and various new participatory/co-creation approaches to democracy. (This is just a suggestion though, not a strict requirement for the acceptance of this paper.)

 In the following, I offer some more specific comments.

 Page 6: It would be interesting to explore “democracy as a feature and property of resilience” vs. “resilience as a feature of democracy”. I actually think the two are closely related: decentralized bottom-up elements and collective intelligence-based approaches are features of democracies, which are also significant for resilience. The same applies to diversity/pluralism and to a fair competition of ideas (“the best approach should win”).

 It would be further interesting to learn what is known about the transition of democracies to autocratic systems? What are the drivers of this process? Identifying them would allow one to defend against this process more effectively. I think that democracies are not only degrading. They are under attack, for example, by hybrid war and, potentially, by surveillance capitalism as well. They are also being questioned or challenged by various hierarchical institutions and platform monopolies.

 Page 10: The second paragraph is mentioning the role of social media platforms, which may support authoritarianism. In fact, this very much depends on the network structure, which has become more centralized over time. Recent publications, for example, have stressed the role of network structure for the diversity of opinions in a system.

 Page 11 mentions the role of private platform ownership. It may be mentioned more explicitly that these are “hijacking” attention by means of big nudging/micro-targeting, by auctions of human attention.

 The limitations of fact-checking should be mentioned explicitly. It is slow, while microtargeting is automated. Moreover, it cannot cope with manipulation based on converging technologies.

 Page 13: “there can also be democratic forms of fascism”: I think this formulation is highly misleading. The sentence probably wanted to articulate that some current forms of democracies have been hollowed out and their cores have been replaced by elements of fascism.

 Democratic ideals: Be more concrete in what you mean: constitutional values, human rights?

 You mention “collective problem-solving” in the last paragraph. It may be worth mentioning the role that “collective intelligence” can play in thriving democracies, if suitable participatory frameworks are offered for this.

**Minor:**

 Page 4: I would suggest to replace “the delaminates” by “that delimits”

 “As an act of resistance, resilient action is always a political and normative form of action.”: I do not share this view. It appears too special/restrictive to me. You may find more general definitions in the literature on “participatory resilience”.

 “But neither resistance nor resilience or automatically…” should probably be replaced by “But neither resistance nor resilience are automatically…”

 Page 5: “… systems are actors and systems, not humans,…” might be better replaced by “… systems are actors and systems. These systems, not humans,…”

 “the present author takes an actor- and action-based human-oriented approach…”: What about the widespread role of AI in today’s society? It should probably be considered as well (e.g. in connection with mis- and disinformation, opinion manipulation).

 “we can distinguish between environmental, economic, political, and cultural crisis”: “technological” is missing, plus the close interrelationship of all of these areas in the information society, so we are actually faced with “techno-socio-economic-environmental systems”

 I would suggest to replace “equity and diversity” by “fairness and diversity”.

 Page 7: In Table 1, I would replace “economic organisations” by “economic institutions”

 Why do you start columns in your various Tables typically with capital letters – despite in column 3?

 Table 2: Shouldn’t “terrorism and organized crime” be added and “hybrid warfare” as well?

 What about processes that may slowly undermine the values and principles of democracies?

 What about migration-related risks or health risks (such as pandemics)?

 Page 9: “can be disrupted by environmental, technological economic…”: What about digital systems and processes?

 As much as a “non-commerical”, “data minimization” and “green computing” approach are desirable: are these approaches realistic, as long as surveillance capitalism and hybrid wars reign? How can we regain agency?

 Table 3: “Decentralised data storage” could be better replaced by the more general term “Decentralisation” (everywhere in the manuscript)

 The 3 ^rd^ and 5 ^th^ row (Economy and Culture) have identical entries in the 3 ^rd^ column (probably by accident?)

 “digital giants” would typically be referred to as “big tech” (should be replaced everywhere throughout the entire manuscript, if considered fitting)

 “Political disruption means that digital democracy platforms are censored”: This can be misinterpreted, as the headline suggests you are talking about “resilient digital democracy”, and censored systems would likely not be considered democratic systems

 Page 10: “number of legal rules” may be replaced by “number of legal acts”

 Page 11: “Between November 2023 and November.”: sentence is incomplete.

 Page 12: Besides Wikipedia, OpenStreetMap is another well-known and impressive example of non-commercial participatory platforms.

 Page 13: “decentralized data storage” would be better replaced by the more general term “decentralisation”

Is the argument persuasive and supported by appropriate evidence?

Partly

Is the work clearly and cogently presented?

Yes

Is the topic of the essay discussed accurately in the context of the current literature?

Yes

Does the essay contribute to the cultural, historical, social understanding of the field?

Yes

Reviewer Expertise:

NA

I confirm that I have read this submission and believe that I have an appropriate level of expertise to confirm that it is of an acceptable scientific standard, however I have significant reservations, as outlined above.

# Response

**Published:** 2026-01-30

**Authors:** Christian Fuchs

Response to review 1 , by Christian Fuchs (author)

**Author:**

 Thank you for the constructive review, which has helped me to improve the article. I created a new version that built in your review comments and a second review comment.

**Reviewer:**

 “Overall, I have read the contribution with interest, and consider it worthy of indexing, but considering the comments below in a revision would further strengthen the manuscript. The arguments provided add nicely to the recent discussion on digital democracy/ies. However, the literature appears to be unnecessarily restricted to political science references, while significant contributions have also been recently published in neighboring fields such as computational social science. It would be valuable to mention some of those activities as well. The same applies to participatory resilience and various new participatory/co-creation approaches to democracy. (This is just a suggestion though, not a strict requirement for the acceptance of this paper.)”

**Author:**

 I have added a discussion of three highly-cited works about disinformation and fake news that utilise computational social sciences, which resulted in the paragraph that starts with “Given the rise of new threats to (digital) democracy, such as fake news, Lazer et al. (2018) called for the creation of a “science of fake news”,….”. One can discuss what studies to present here, but this is a matter of choice and one cannot mention everything. I also want to point out that my paper is conceptual in nature and wants to introduce and theorise the notion of resilient digital democracy. **Reviewer:**

 “Page 6: It would be interesting to explore “democracy as a feature and property of resilience” vs. “resilience as a feature of democracy”. I actually think the two are closely related: decentralized bottom-up elements and collective intelligence-based approaches are features of democracies, which are also significant for resilience. The same applies to diversity/pluralism and to a fair competition of ideas (“the best approach should win”).”

**Author:**

 The point I want to make in this paragraph is that the term “democratic resilience” can be confusing. I added the following passage: “Democratic resilience is an organisational mode of resilience. There can be potentially other non-democratic forms of organising resilience.  In this paper, we are interested in how digital democracy can be made resilient and not so much in resilience’s organizational mode, which is why therefore we avoid using the term “democratic resilience” and prefer to speak of “resilient democracy” and “the resilience of democracy”.”

 I do not feel able to expand on this topic and think it is not central for conceptualising resilient digital democracy. 

**Reviewer:**

 “It would be further interesting to learn what is known about the transition of democracies to autocratic systems? What are the drivers of this process? Identifying them would allow one to defend against this process more effectively. I think that democracies are not only degrading. They are under attack, for example, by hybrid war and, potentially, by surveillance capitalism as well. They are also being questioned or challenged by various hierarchical institutions and platform monopolies.”

**Author:**

 I have added something on this topic to the introduction, namely the following passage:

 "In the academic literature, one finds a distinction between three processes of autocratisation, namely “democratic regression/backsliding (when the core features of liberal democracy are eroded but no transition to autocratic regime occurs), democratic breakdown (where democracy collapses), or authoritarian deepening (when we observe the deepening of authoritarian characteristics in already autocratic regimes)“ (Tomini, Gibril & Bochev 2023, 120). Waldner and Lust (2018) review theories that explain the causes of the rise of authoritarianism. They distinguish between “six theory families” explaining autocratisation (109): There are approaches that stress the role of political actors, political culture, political institutions, political economy (income levels, income distribution, macroeconomic development, class relations, etc.), conflicts among groups of citizens, and international actors. Waldner and Lust argue for a model that synthesises such explanations. The six theory families include economic, political, and cultural factors. If a synthesis is feasible, then the implication is that there can be economic, political, and cultural factors that make the rise or strengthening of authoritarianism more likely. In a comparable manner, the political theorist Franz L. Neumann (2017) argues that there are economic, political, and socio-cultural forms of alienation that drive the rise or intensification of authoritarianism if they combine with massive fears among the population (destructive psychological alienation) that turns into persecutory anxiety organised by authoritarian parties, leaders, institutions, or social movements (see also Fuchs 2017)."

**Reviewer:** Page 10: The second paragraph is mentioning the role of social media platforms, which may support authoritarianism. In fact, this very much depends on the network structure, which has become more centralized over time. Recent publications, for example, have stressed the role of network structure for the diversity of opinions in a system.

**Author:**

 I agree, so I changed the formulation that platforms “make democratic information and communication prone to political and ideological disruption strategies“ a bit weaker, saying “can meke …“ I must say that find review comments that speak of “recent publications”, “contributions have also been recently published”, etc. without mentioning and pointing towards specific papers not helpful because they remain too abstract. At the same time, I think a reviewer not indicating such sources should not do so in a later review because it was his/her mistake not to mention sources one could refer to in the first instance.

**Reviewer:** Page 11 mentions the role of private platform ownership. It may be mentioned more explicitly that these are “hijacking” attention by means of big nudging/micro-targeting, by auctions of human attention.

**Author:**

 That’s a good and important point so I added it (“digital capital, digital giants and digital monopolies that use strategies such as big data collection, micro-targeting, algorithmic analysis for hijacking and managing users’ attention, see Fuchs 2024”)

**Reviewer:**

 The limitations of fact-checking should be mentioned explicitly. It is slow, while microtargeting is automated. Moreover, it cannot cope with manipulation based on converging technologies.

**Author:**

 That’s a good and important point. I now pointed towards the problems of fact-checking:

 “Fact-checking organisations require adequate resources, including funding, fact-checking technologies, and highly educated and well-paid fact-checking professionals. They face the problem that the creation and dissemination of fake news and disinformation operates at high speed and is partly or fully automated while fact-checking requires significant amounts of labour-time. Therefore, the issue arises of how to best manage the antagonism between the high speed of fake news and the slow speed of fact-checking. Fact-checking needs to be combined with other measures such as the creation of alternative, non-commercial, democratic Internet platforms.”

**Reviewer:**

 Page 13: “there can also be democratic forms of fascism”: I think this formulation is highly misleading. The sentence probably wanted to articulate that some current forms of democracies have been hollowed out and their cores have been replaced by elements of fascism.

**Author:**

 I added a clarification:

 “However, there can also be what Amlinger and Nachtwey (2025) term “democratic fascism” where democracy is used to advance fascism, which does not mean that fascism is democratic, but that fascism uses the structures, concepts, rhetoric, and language of democracy to present itself as democratic and democrats as undemocratic in order to successively undermine democracy from within. Lührmann and Lindberg (2019) identified 217 instances of autocratisation in a total of 109 countries in the years from 1900 until 2017. They speak of three waves of autocratisation (1926-1942, 1961-1977, since 1994). The third wave corresponds to what Amlinger and Nachtwey describe as democratic fascism, fascism’s hijacking of democracy: “Democratic erosion became the modal tactic during the third wave of autocratization. Here, incumbents legally access power and then gradually, but substantially, undermine democratic norms without abolishing key democratic institutions. Such processes account for 70% in the third reversal wave with prominent cases of such gradual deterioration“ (Lührmann and Lindberg 2019, 1105).The implication of such developments is that public and commons projects need a deliberate commitment to democracy”

**Reviewer:**

 Democratic ideals: Be more concrete in what you mean: constitutional values, human rights?

**Author:** I added a short explanation

**Reviewer:**

 You mention “collective problem-solving” in the last paragraph. It may be worth mentioning the role that “collective intelligence” can play in thriving democracies, if suitable participatory frameworks are offered for this.

**Author:**

 I am not a big fan of the notion and term of collective intelligence coined by Pierre Levy or related terms such as the wisdom of the crowd, so I did not add it.

**Various minor points the reviewer mentioned:**

**Author:**

 Thank you, these were typos and other minor issues, I fixed them according to your guidance

**Reviewer:**

 “the present author takes an actor- and action-based human-oriented approach…”: What about the widespread role of AI in today’s society? It should probably be considered as well (e.g. in connection with mis- and disinformation, opinion manipulation).

**Author:**

 That’s a good point, I added a new paragraph on this topic:

 “An action-based humanist approach faces the question if autonomous AI-based system such as self-driving cars, autonomous weapon systems, cleaning robots, generative AI systems such as ChatGPT, or Internet bots are actors or not. From a humanist perspective, the answer is that so-called autonomous technologies do not operate independently from humans but depend on them. They depend on human engineering, programming, repair, energy produced by humans, etc. In addition, they do not have the same features as humans have, which is why robots and AI cannot replace humans. “Robots cannot truly replace humans because they lack praxis and poiesis, which means they do not understand what it means to be social, what it means to love, what it means to be sad, what morality is, what desires and creativity are etc. They lack fundamental human capacities. Robots do not have feelings. They do not have intentions. They do not have morals. They do not have desires. They do not have a will. They do not understand and experience love, sadness and happiness. Computers cannot deal with ambiguity and dialectics. They operate based on algorithms, while humans are non-algorithmic” (Fuchs 2022, 146-147). The philosopher Hubert Dreyfus (1986, xiv) argues in this context: “Human emotional life remains unique, to be sure, but what is more important is our ability to recognize, to synthesize, to intuit. There are good reasons to believe that those abilities as well are rooted in processes altogether different from the calculative reason of computer programs”.”

**Reviewer:**

 “we can distinguish between environmental, economic, political, and cultural crisis”: “technological” is missing, plus the close interrelationship of all of these areas in the information society, so we are actually faced with “techno-socio-economic-environmental systems”

**Author:**

 I changed this sentence and added something on this topic:

 we can distinguish between environmental, technological, economic, political, and cultural crises (see table 1). Hofkirchner (2002, 2006) adds that society consists of the sociosphere, the technosphere, and the ecosphere. He divides the sociosphere into the economy, the political system, and culture.

**Reviewer:**

 I would suggest to replace “equity and diversity” by “fairness and diversity”.

**Author:**

 That’s a direct quote so it cannot be replaced…

**Reviewer:**

 Table 2: Shouldn’t “terrorism and organized crime” be added and “hybrid warfare” as well?

 What about processes that may slowly undermine the values and principles of democracies?

 What about migration-related risks or health risks (such as pandemics)?

**Author:**

 I amended the table…

**Reviewer:**

 Page 9: “can be disrupted by environmental, technological economic…”: What about digital systems and processes?

**Author:**

 “digital” is part of “technological”

**Reviewer:**

 As much as a “non-commerical”, “data minimization” and “green computing” approach are desirable: are these approaches realistic, as long as surveillance capitalism and hybrid wars reign? How can we regain agency?

**Author:**

 the point is that we have to overcome surveillance capitalism and hybrid wars in order to establish such alternatives as dominant forms, but the problem is that Europe has not created enough alternative Internet platforms and technologies, and it can still start to do so, that’s my key point, it is never too late.

**Reviewer:**

 Page 12: Besides Wikipedia, OpenStreetMap is another well-known and impressive example of non-commercial participatory platforms.

**Author:**

 A very good point that I added …

 “Others include, for example, OpenStreetMap (run by the OpenStreetMap Foundation), Creative Commons, Wayback Machine (run by the Internet Archive), or FLOSS (free/libre/open source software)”.

**Author:**

 Overall, the review has much helped me to improve the paper’s quality.
